# A general framework for powerful confounder adjustment in omics association studies

**DOI:** 10.1093/bioinformatics/btad563

**Published:** 2023-09-09

**Authors:** Asmita Roy, Jun Chen, Xianyang Zhang

**Affiliations:** epartment of Statistics, Texas A&M University, 155 Ireland Street, College Station, TX 77840, United States; Division of Computational Biology, Mayo Clinic, 200 1st St. SW, Rochester, MN 55905, United States; epartment of Statistics, Texas A&M University, 155 Ireland Street, College Station, TX 77840, United States

## Abstract

**Motivation:**

Genomic data are subject to various sources of confounding, such as demographic variables, biological heterogeneity, and batch effects. To identify genomic features associated with a variable of interest in the presence of confounders, the traditional approach involves fitting a confounder-adjusted regression model to each genomic feature, followed by multiplicity correction.

**Results:**

This study shows that the traditional approach is suboptimal and proposes a new two-dimensional false discovery rate control framework (2DFDR+) that provides significant power improvement over the conventional method and applies to a wide range of settings. 2DFDR+ uses marginal independence test statistics as auxiliary information to filter out less promising features, and FDR control is performed based on conditional independence test statistics in the remaining features. 2DFDR+ provides (asymptotically) valid inference from samples in settings where the conditional distribution of the genomic variables given the covariate of interest and the confounders is arbitrary and completely unknown. Promising finite sample performance is demonstrated via extensive simulations and real data applications.

**Availability and implementation:**

R codes and vignettes are available at https://github.com/asmita112358/tdfdr.np.

## 1 Introduction

One central theme of genomic data analysis is identifying genomic features associated with a variable of interest, such as disease status. Due to the constraint of clinical sample collection, the variable of interest is often correlated with other variables, which may confound the associations of interest. For example, when identifying microbiome biomarkers for endometrial cancer, the age of patients acts as a confounder, as older patients tend to have more malignant tumors, and the female reproduction tract microbiome changes with age. Controlling for confounders is crucial for successful validation, cost reduction, and faster translation of discoveries to clinical tests. However, confounder adjustment in genome-scale association tests exacerbates the low statistical power and inflates the type I error rate.

The traditional way of confounder adjustment for high-dimensional association tests is to adjust for confounders for each genomic feature and correct the individual association *P*-values for multiple testing using FDR control (Benjamini and Hochberg 1995, Storey 2002). However, adjusting confounders for every feature can lead to power loss when confounders only affect a subset of features. To address this, a two-dimensional false discovery rate control procedure (2DFDR) based on linear models was proposed Yi *et al.* (2021). The 2DFDR procedure screens out irrelevant features using unadjusted statistics (from fitting the unadjusted linear models to each omics feature) and identifies true signals using adjusted (from fitting the confounder-adjusted linear models to each omics feature) statistics, controlling the FDR at the desired level.

Although 2DFDR is an improvement over previous methods, its implementation has limitations. It is only applicable to normally distributed outcomes, while omics studies generate different outcome types. Extending 2DFDR to handle binary or discrete outcomes is desired. Additionally, type I error inflation can occur in certain scenarios, particularly with high confounding effects or a small number of features. Improving the type I error control of 2DFDR in these cases would enhance its robustness and reliability.

To address these limitations, we propose a general framework called 2DFDR+ for integrating confounder adjustment into multiple testing. The 2DFDR+ framework extends the original 2DFDR in several aspects.

1. It relaxes the linear model assumption of Yi *et al.* (2021), accommodating various outcome types such as continuous, binary, and count outcomes.
2. The marginal test statistic acts as an auxiliary statistic in this method, which improves power by screening out noise.
3. It provides a unified approach to approximate the joint distribution of test statistics under the null hypothesis, eliminating the need for the case-by-case derivation of the joint (asymptotic) distribution of the conditional and marginal independence test statistics of 2DFDR.
4. It enables different methods for estimating the conditional distribution of the covariate given the confounders, including permutation/bootstrap, simulation, MCMC, and conditional GAN.
5. It improves FDR control by explicitly modeling the relationship between the variable of interest and confounders, especially in the presence of strong confounding effects.

Theoretical analysis demonstrates that 2DFDR+ provides asymptotic FDR control and retains the same number of rejections as the corresponding 1D procedure. A unique feature of 2DFDR+ is that it lets the data decide the usefulness/informativeness of the auxiliary statistic. If the auxiliary statistic provides helpful information, 2DFDR+ has significant power gain, otherwise, it reduces to the corresponding 1D procedure. When the FDR is controlled at level *q*, we can show that in the worst-case scenario, the asymptotic power loss for 2DFDR+ compared to the 1D procedure is at most *q*, see Supplementary Section S1.

Section 2 describes the problem setup and a two-dimensional (2d) rejection region based on a primary statistic for testing the conditional independence between the omics feature and the covariate of interest given the confounders and an auxiliary statistic for testing the marginal independence between the omics feature and the covariate. Section 3 introduces an oracle FDR-controlling procedure, where the conditional distribution of the covariate given the confounders is assumed to be known. Sections 5 and 6 are devoted to numerical studies and real data analyses, respectively.

## 2 Problem statement and 2d rejection region

We formulate the feature selection problem by allowing the omics variables to depend on the covariate of interest and confounders arbitrarily. To state the problem and the procedure carefully, suppose we have *n* i.i.d. samples ${\left\{(X_{i},Y_{i},Z_{i})\right\}}_{i=1}^{n}$ with $Y_{i}={\left(Y_{i,1},\ldots ,Y_{i,m}\right)}^{⊤}$ from a population, each of the form (X,Y,Z), where $X\in R^{p},\;Y={\left(Y_{1},\ldots ,Y_{m}\right)}^{⊤}\in R^{m}$ and $Z={\left(Z_{1},\ldots ,Z_{d}\right)}^{⊤}\in R^{d}$. Here Y represents a vector of omics features, X is the covariate of interest, and Z denotes the set of confounders. We aim to discover as many as possible omics features *Y_i_* that are dependent of X conditionally on the confounders Z. We formulate this as the problem of testing

H0,j:Yj⊥⊥X|Z against H1,j:Yj⊥⊥X|Z

for 1≤j≤m. To tackle this problem, one must adjust for the confounders and the multiplicity in testing. The burden from both adjustments could lead to potential power loss, especially when the confounding effect is strong.

Our idea to resolve this issue is to use two statistics jointly, namely a primary statistic for testing the conditional independence specified in $H_{0,j}$ and an auxiliary statistic for testing the marginal independence $Y_{j}⊥⊥X$, for deciding whether or not to reject $H_{0,j}$. The purpose of using the auxiliary statistic is to enrich signals, reduce the multiple testing burden, and thus enhance the multiple testing power. As marginal dependence does not necessarily imply conditional dependence (e.g. *Y_j_* and X are both functions of Z), the use of auxiliary statistics could lead to selection bias and requires proper adjustment in the selection of cut-off values. One of our goals is to carefully design a way to simultaneously select the cut-off values for the primary statistic and the auxiliary statistic to control the FDR at the desired level.

As a motivation, we consider *m* independent generalized linear models:

$$\begin{array}{c} f(Y_{j}|X,Z,\alpha_{j},\beta_{j},\phi_{j})=\text{exp}\;\left\{\frac{\theta_{j}Y_{j}-b(\theta_{j})}{\phi_{j}}+c(Y_{j},\phi_{j})\right\},\\ g(E[Y_{j}])=g(b\prime (\theta_{j}))=X^{⊤}\alpha_{j}+Z^{⊤}\beta_{j}, \end{array}$$

for 1≤j≤m, where *g* is a known link function, *θ_j_* is the canonical parameter, $\phi_{j}$ is the dispersion parameter, $c(Y_{j},\phi_{j})$ is some function of $\left(Y_{j},\phi_{j}\right)$ and $\alpha_{j}\in R^{p},\beta_{j}\in R^{d}$ are the coefficients associated with the covariate of interest and confounders, respectively. Under the above model, there are four different categories to consider as follows:

- Associated with both the covariate of interest and confounders: $\alpha_{j}\neq 0,\beta_{j}\neq 0;$
- Solely associated with the covariate of interest: $\alpha_{j}\neq 0,\beta_{j}=0$;
- Solely associated with the confounders: $\alpha_{j}=0,\beta_{j}\neq 0$;
- Not associated with either the covariate of interest or confounders: $\alpha_{j}=0,\beta_{j}=0.$

We note that: (i) $\alpha_{j}=0$ if and only if $Y_{j}⊥⊥X|Z$; (ii) when $\beta_{j}=0$ (Categories B and D), testing the conditional independence boils down to testing the marginal independence $Y_{j}⊥⊥X$. In a general setting, these four categories can be described as: (A) Yj⊥⊥X|Z and Yj⊥⊥Z|X; (B) $Y_{j}⊥⊥Z|X$ and Yj⊥⊥X; (C) $Y_{j}⊥⊥X|Z$ and Yj⊥⊥Z; (D) $Y_{j}⊥⊥(X,Z)$. As a way to enrich signals, we use a marginal independence test to screen out the omics features in Category D and further use a conditional independence test to pick out the true signals from Categories A and B. More precisely, we let $T_{j}^{C}$ and $T_{j}^{M}$ be two test statistics computed based on the samples ${\left\{(X_{i},Y_{i},Z_{i})\right\}}_{i=1}^{n}$ for testing the conditional independence $Y_{j}⊥⊥X|Z$ and the marginal independence $Y_{j}⊥⊥X$, respectively. Throughout the discussions below, we assume that a large positive value of $T_{j}^{M}$ ($T_{j}^{C}$) provides evidence against marginal (conditional) independence. The readers are referred to Supplementary Section S4 for some examples of conditional and unconditional independence tests. Given the thresholds, $t_{1},t_{2}\geq 0$, the 2d procedure can be described as follows.

Dimension 1. Use the marginal independence test statistics to determine a preliminary set of features $D_{1}=\left\{1\leq j\leq m:T_{j}^{M}\geq t_{1}\right\}$.

Dimension 2. Reject $H_{0,j}$ for $T_{j}^{C}\geq t_{2}$ and $j\in D_{1}$. As a result, the final set of discoveries is given by $D_{2}=\left\{1\leq j\leq m:T_{j}^{M}\geq t_{1},T_{j}^{C}\geq t_{2}\right\}$.

Although marginal dependence does not imply conditional dependence, it can be leveraged to increase the signal density and reduce multiple testing burden in the second dimension. More precisely, the usefulness of the marginal dependence test is due to

1. the marginal dependence test statistics screen out a large number of noises in Category D and thus ease the multiple testing burden in the second dimension;
2. the marginal dependence test statistics are more effective in detecting signals from Category B as the conditional dependence test causes over-adjustment, reducing the signal strength.

We illustrate the rational behind 2DFDR+ through the following example. A detailed description of the method is provided in the next section.

**Figure 1.**
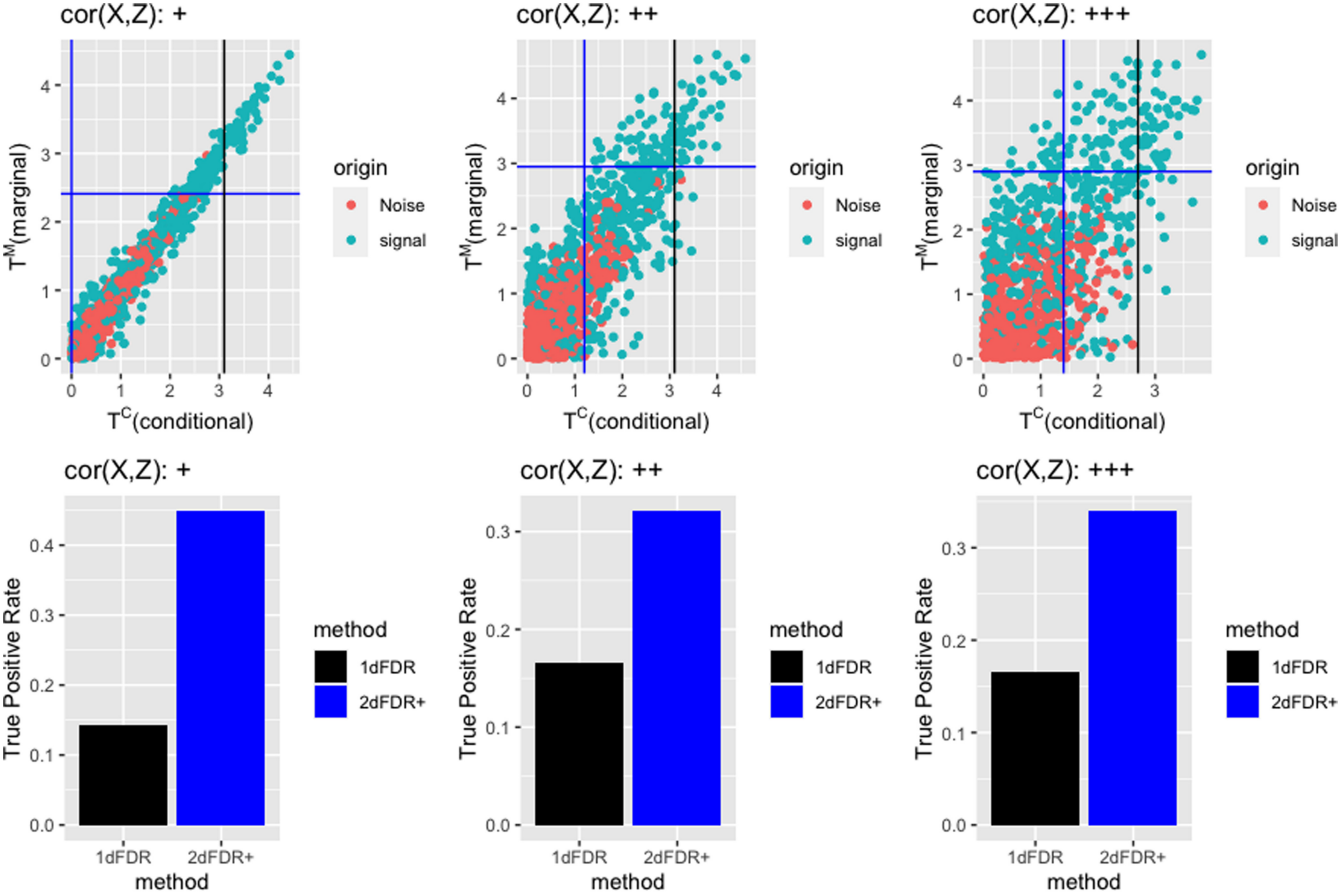
Illustration of 2dFDR+ using simulated datasets. The three panels in the first row denote the decision boundaries for 1dFDR and 2dFDR+ at the 5% FDR level for three degrees of confounding. 1dFDR relies on the conditional statistic (*T^C^*) only (one dimension) while 2dFDR+ is based on both the marginal and the conditional statistics (*T^C^* and *T^M^*), i.e. it uses two dimensions, leading to significant power gain

Example 1. Consider the following data generating process:

(1) $$Y_{j}∼\text{Bernoulli}(p_{j}),\;\text{log}\;\left(\frac{p_{j}}{1-p_{j}}\right)=\alpha_{j}X+\beta_{j}Z,$$

where $X=(\rho Z+\epsilon )/\sqrt{\rho^{2}+1}$ with *Z* and ϵ∼N(0,1), independently. ρ∈{0.1,0.5,1} represents weak (+), medium (++), and strong (+++) confounding levels. $\alpha_{j},\beta_{j}∼0.15\times \text{Unif}(-0.7,-0.5)+0.15\times \text{Unif}(0.5,0.7)+0.7\times \delta_{0},$ independently, where *δ*_0_ denotes a point mass at 0. $T_{j}^{C}$ is the *t*-statistic for testing ${\tilde{H}}_{0,j}:\alpha_{j}=0$ under the logistic model (1), and $T_{j}^{M}$ is the *t*-statistic for testing ${\tilde{H}}_{0,j}$ under the reduced model by forcing $\beta_{j}=0$ in model (1). In Fig. 1, we plot the marginal (*T^M^*) statistic against the conditional (*T^C^*) statistic for various confounded scenarios. The standard approach performs (1D) FDR control based on the conditional statistic (*T^C^*) only (we refer it as 1DFDR). When the correlation between the variable of interest and the confounder (denoted as $\text{cor}(X,Z)=\rho /\sqrt{\rho^{2}+1}\in \{0.1,0.45,0.71\}$) is high, the signals (green) and noises (red) overlap much on *T^C^*. To achieve the desired FDR level, 1DFDR requires a high cutoff (black line). For 2DFDR+, it first uses *T^M^* to exclude a large number of irrelevant features (horizontal blue line). Next, a lower cutoff (vertical blue line) is used to achieve the same FDR level. As a result, it achieves significant power improvement, and the improvement increases with the correlation between the variable of interest and the confounder.

## 3 Oracle procedure

We introduce an oracle FDR-controlling procedure, where we assume that the conditional distribution of X given Z, denoted by $P_{X|Z}$ below, is known. Supplementary Section S3 introduces several ways of estimating this conditional distribution from the observations.

### 3.1 Estimating the false discovery proportion

Our goal here is to develop a principled way of finding the cutoff values (*t*_1_, *t*_2_) such that the FDR is controlled at a desired level while the number of rejections is as large as possible. Let $M_{0}=\{1\leq j\leq m:H_{0,j}\;\text{is true}\}$ and $m_{0}=|M_{0}|$ be the set and the number of true null hypotheses, respectively. Write $\tilde{X}={\left(X_{1},\ldots ,X_{n}\right)}^{⊤},\;{\tilde{Y}}_{j}={\left(Y_{1,j},\ldots ,Y_{n,j}\right)}^{⊤}$ and $\tilde{Z}={\left(Z_{1},\ldots ,Z_{n}\right)}^{⊤}.$ Based on the 2d rejection region, the false discovery proportion (FDP) is given by

(2) $$\text{FDP}(t_{1},t_{2})=\frac{\sum_{j\in M_{0}}1\{T_{j}^{M}\geq t_{1},T_{j}^{C}\geq t_{2}\}}{1\vee \sum_{j=1}^{m}1\{T_{j}^{M}\geq t_{1},T_{j}^{C}\geq t_{2}\}},$$

where a∨b=max{a,b} for a,b∈R. Note that the FDP is zero when no rejection is made. We replace the numerator in the definition of $\text{FDP}(t_{1},t_{2})$ by its conditional expectation with respect to $\tilde{X}$ given ${\tilde{Y}}_{j}$ and $\tilde{Z}$, which leads to the following approximate upper bound on the FDP:

(3) $$\begin{array}{c} \text{FDP}(t_{1},t_{2})\approx \frac{\sum_{j\in M_{0}}P_{0}(T_{j}^{M}\geq t_{1},T_{j}^{C}\geq t_{2}|{\tilde{Y}}_{j},\tilde{Z})}{1\vee \sum_{j=1}^{m}1\{T_{j}^{M}\geq t_{1},T_{j}^{C}\geq t_{2}\}}\\ \leq \frac{\sum_{j=1}^{m}P_{0}(T_{j}^{M}\geq t_{1},T_{j}^{C}\geq t_{2}|{\tilde{Y}}_{j},\tilde{Z})}{1\vee \sum_{j=1}^{m}1\{T_{j}^{M}\geq t_{1},T_{j}^{C}\geq t_{2}\}} \end{array}$$

(4) $$:={\text{FDP}}_{\text{oracle}}(t_{1},t_{2}),$$

where $P_{0}(\cdot |{\tilde{Y}}_{j},\tilde{Z})$ denotes the conditional probability under the null hypothesis $H_{0,j}$. The upper bound ${\text{FDP}}_{\text{oracle}}(t_{1},t_{2})$ relies on the conditional distribution $P_{X|Z}$. To find a feasible conservative estimator of the FDP, it remains to estimate the conditional probabilities in the numerator of ${\text{FDP}}_{\text{oracle}}(t_{1},t_{2})$. To this end, we write $T_{j}^{M}=T_{j}^{M}(\tilde{X},{\tilde{Y}}_{j})$ and $T_{j}^{C}=T_{j}^{C}(\tilde{X},{\tilde{Y}}_{j},\tilde{Z})$ to emphasize their dependence on the samples. As $P(\tilde{X}|{\tilde{Y}}_{j},\tilde{Z})=P(\tilde{X}|\tilde{Z})$ under $H_{0,j}$ and $P(\tilde{X}|\tilde{Z})=\prod_{i=1}^{n}P_{X|Z}(X_{i}|Z_{i})$, we have under $H_{0,j}$ that

$$\begin{array}{l} P_{0}(T_{j}^{M}(\tilde{X},{\tilde{Y}}_{j})\geq t_{1},T_{j}^{C}(\tilde{X},{\tilde{Y}}_{j},\tilde{Z})\geq t_{2}|{\tilde{Y}}_{j},\tilde{Z})\\ =E\left[1\{T_{j}^{M}(\tilde{X},{\tilde{Y}}_{j})\geq t_{1},T_{j}^{C}(\tilde{X},{\tilde{Y}}_{j},\tilde{Z})\geq t_{2}\}|{\tilde{Y}}_{j},\tilde{Z}\right]\\ =\int 1\{T_{j}^{M}(\tilde{x},{\tilde{Y}}_{j})\geq t_{1},T_{j}^{C}(\tilde{x},{\tilde{Y}}_{j},\tilde{Z})\geq t_{2}\}d\prod_{i=1}^{n}P_{X|Z}(x_{i}|Z_{i}), \end{array}$$

where $\tilde{x}={\left(x_{1},\ldots ,x_{n}\right)}^{⊤}$ with $x_{i}\in R^{p}$, which can be calculated once we know the conditional distribution $P_{X|Z}$. One way to approximate $P_{0}(T_{j}^{M}\geq t_{1},T_{j}^{C}\geq t_{2}|{\tilde{Y}}_{j},\tilde{Z})$ is via Monte Carlo simulation. Specifically, we generate $X_{i,b}{∼}^{\text{ind}}P_{X|Z}(\cdot |Z_{i}),\;i=1,2,\ldots ,n,\;\;b=1,2,\ldots ,B.$ In practice, this distribution is unknown. Details on how to estimate this can be found in Supplementary Section S3. Denote by $T_{j,b}^{M}$ and $T_{j,b}^{C}$ the marginal and conditional independence test statistics computed based on $\left({\tilde{X}}_{b},{\tilde{Y}}_{j},\tilde{Z}\right)$ with ${\tilde{X}}_{b}={\left(X_{1,b},\ldots ,X_{n,b}\right)}^{⊤}$, respectively. We propose to estimate $P_{0}(T_{j}^{M}\geq t_{1},T_{j}^{C}\geq t_{2}|{\tilde{Y}}_{j},\tilde{Z})$ by

$${\bar{F}}_{j,B}(t_{1},t_{2}):=\frac{1}{B+1}\sum_{b=0}^{B}1\{T_{j,b}^{M}\geq t_{1},T_{j,b}^{C}\geq t_{2}\}$$

with $\left(T_{j,0}^{M},T_{j,0}^{C})=(T_{j}^{M},T_{j}^{C}\right)$. Hence a conservative estimate for the FDP is given by

$$\tilde{\text{FDP}}(t_{1},t_{2})=\frac{\sum_{j=1}^{m}{\bar{F}}_{j,B}(t_{1},t_{2})}{1\vee \sum_{j=1}^{m}1\{T_{j}^{M}\geq t_{1},T_{j}^{C}\geq t_{2}\}}.$$

### 3.2 Finding the optimal cut-off

We now introduce a greedy approach to select the cut-offs. For a desired FDR level *q*, we first define

$$F_{q}=\{(t_{1},t_{2})\in R^{+}\times R^{+}:\tilde{\text{FDP}}(t_{1},t_{2})\leq q\}$$

as the feasible set that contains all the cut-off values controlling the FDP estimate at the level *q*. We then select the optimal cut-off as the one delivering the most number of rejections from the feasible set:

$$(t_{1}^{*},t_{2}^{*})=\underset{(t_{1},t_{2})\in F_{q}}{\text{argmax}}\sum_{j=1}^{m}1\{T_{j}^{M}\geq t_{1},T_{j}^{C}\geq t_{2}\}.$$

Finally, we reject all the hypotheses $H_{0,j}$ such that

$$T_{j}^{M}\geq t_{1}^{*}\;\;\text{and}\;\;T_{j}^{C}\geq t_{2}^{*}.$$

A summary of the method can be found in Algorithm 1. We remark that the parameter *ν* controls the searching accuracy of the procedure.

Remark 1. In the Supplementary Material, we describe a variant of the 2d procedure (2d-FWER+) to control the family-wise error rate (FWER). Simulation studies suggest that 2d-FWER+ provides reliable FWER control in finite sample.

### 3.3 Estimating the null proportion

Following the idea in Storey (2002), we can further improve the power of our method by estimating the proportion of null hypotheses. As a motivation, we suppose $T_{j}^{C}$ follows the mixture distribution $\pi_{0}P_{0}+(1-\pi_{0})P_{1},$ where *π*_0_ represents the null proportion, $P_{0}$ and $P_{1}$ denote the distributions under the null and alternative, respectively. Under this two-group mixture model, we have $P(T_{j}^{C}\leq \lambda )=\pi_{0}P_{0}(T_{j}^{C}\leq \lambda )+(1-\pi_{0})P_{1}(T_{j}^{C}\leq \lambda )\geq \pi_{0}P_{0}(T_{j}^{C}\leq \lambda )$, which implies that

$$\frac{\sum_{j=1}^{m}1\{T_{j}^{C}\leq \lambda \}}{\sum_{j=1}^{m}P_{0}(T_{j}^{C}\leq \lambda )}\approx \frac{\sum_{j=1}^{m}P(T_{j}^{C}\leq \lambda )}{\sum_{j=1}^{m}P_{0}(T_{j}^{C}\leq \lambda )}\geq \pi_{0},$$

where the approximation is due to the law of large numbers. Therefore, we propose to estimate the null proportion *π*_0_ by

$${\hat{\pi }}_{0}(\lambda )=1\wedge \frac{\sum_{j=1}^{m}1\{T_{j}^{C}\leq \lambda \}}{\sum_{j=1}^{m}F_{j,B}(\lambda )},\;F_{j,B}(\lambda ):=\frac{1}{B+1}\sum_{b=0}^{B}1\{T_{j,b}^{C}\leq \lambda \}.$$

We can then implement the 2DFDR+ based on the following estimate of the FDP:

$${\tilde{\text{FDP}}}_{\lambda }(t_{1},t_{2})=\frac{{\hat{\pi }}_{0}(\lambda )\sum_{j=1}^{m}{\bar{F}}_{j,B}(t_{1},t_{2})}{1\vee \sum_{j=1}^{m}1\{T_{j}^{M}\geq t_{1},T_{j}^{C}\geq t_{2}\}},$$

which can be regarded as John Storey’s version of the 2DFDR+ procedure.

Algorithm 1.

1. *Collect samples:* $\tilde{X}={\left(X_{1},\ldots ,X_{n}\right)}^{⊤},\;{\tilde{Y}}_{j}={\left(Y_{1,j},\ldots ,Y_{n,j}\right)}^{⊤}$*and* $\tilde{Z}={\left(Z_{1},\ldots ,Z_{n}\right)}^{⊤}.$
2. *For* 1≤j≤m*, compute the test statistics* $T_{j}^{M}$*and* $T_{j}^{C}$*based on* $\left\{\tilde{X},{\tilde{Y}}_{j},\tilde{Z}\right\}$.
3. ***G****enerate the bootstrap samples* $X_{i,b}{∼}^{\text{ind}}{\hat{P}}_{X|Z}(\cdot |Z_{i})$*for* 1≤i≤n*and* 1≤b≤B*, where* ${\hat{P}}_{X|Z}$*is the estimate of* $P_{X|Z}$.
4. *For* 1≤b≤B*and* 1≤j≤m*, compute the bootstrap test statistics* $T_{j,b}^{M}$*and* $T_{j,b}^{C}$*based on* $\left\{{\tilde{X}}_{b},{\tilde{Y}}_{j},\tilde{Z}\right\}$*with* ${\tilde{X}}_{b}={\left(X_{1,b},\ldots ,X_{n,b}\right)}^{⊤}$.
5. *Let* $T_{\left(j\right)}^{M}$*(and* $T_{\left(j\right)}^{C}$*) be the jth order statistics of* ${\left\{T_{j}^{M}\right\}}_{j=1}^{m}$*(and* ${\left\{T_{j}^{C}\right\}}_{j=1}^{m}$*). Given some integer* 1≤ν≤m*, we define* $S_{1}=\{T_{\left(\left\lfloor i/\nu \right\rfloor \right)}^{M}:i=1,2,\ldots ,\nu \}$*and* $S_{2}=\{T_{\left(\left\lfloor i/\nu \right\rfloor \right)}^{C}:i=1,2,\ldots ,\nu \}$*, where* ⌊a⌋*denotes the integer part of a.*
6. *Compute* $(t_{1}^{*},t_{2}^{*})=\text{argmax}\sum_{j=1}^{m}1\{T_{j}^{M}\geq t_{1},T_{j}^{C}\geq t_{2}\},$*where the maximization is over all* $(t_{1},t_{2})\in S_{1}\times S_{2}$*with* $\hat{\text{FDP}}(t_{1},t_{2})\leq q$*[seeEquation (5) for the definition of*$\hat{\text{FDP}}(t_{1},t_{2})$*]. Reject the jth hypothesis if* $T_{j}^{M}\geq t_{1}^{*}$and $T_{j}^{C}\geq t_{2}^{*}.$

## 4 FDR control

To state the main theorem, we use a list of assumptions defined and justified in the Supplementary Material. The theorem below establishes the asymptotic FDR control of the 2DFDR+ procedure.

Theorem 1. *UnderAssumptions 1–3 in the Supplementary Material and as*B→+∞,

$$\underset{n,m\to +\infty }{\lim\sup}E\left[\text{FDP}(t_{1}^{*},t_{2}^{*})\right]\leq q,$$

*where* $\text{FDP}(t_{1},t_{2})$*is defined inEquation (2).*

In practice, $P_{X|Z}$ is often unknown and has to be estimated from the data; see Supplementary Section S3 for more details. The following result shows that under suitable assumptions on the estimated conditional distribution, ${\hat{P}}_{X|Z}$, the FDR can still be controlled at the target level.

Corollary 1. *Let* ${\hat{F}}_{j,B}(t_{1},t_{2})$*be the corresponding value of* ${\bar{F}}_{j,B}(t_{1},t_{2})$*based on* $\left\{X_{i,b}:1\leq i\leq n,1\leq b\leq B\right\}$*sampled from* ${\hat{P}}_{X|Z}$*. Define*

(5) $$\tilde{\text{FDP}}(t_{1},t_{2})=\frac{\sum_{j=1}^{m}{\hat{F}}_{j,B}(t_{1},t_{2})}{1\vee \sum_{j=1}^{m}1\{T_{j}^{M}\geq t_{1},T_{j}^{C}\geq t_{2}\}}$$

*and the corresponding cutoffs as:*

$$({\hat{t}}_{1},{\hat{t}}_{2})=\underset{(t_{1},t_{2})\in R^{+}\times R^{+}:\hat{\text{FDP}}(t_{1},t_{2})\leq q}{\text{argmax}}\sum_{j=1}^{m}1\{T_{j}^{M}\geq t_{1},T_{j}^{C}\geq t_{2}\}.$$

*Then underAssumptions 1–5 in the Supplementary Material*,

$$\underset{n,m,B\to +\infty }{\lim\sup}E\left[\text{FDP}({\hat{t}}_{1},{\hat{t}}_{2})\right]\leq q.$$

Additional theoretical results on FWER control and power analysis for the algorithm have been relegated to the Supplementary Material.

## 5 Numerical studies

### 5.1 Simulation setting

We conduct comprehensive simulations to evaluate the performance of 2DFDR+ and compare it to competing methods. We generate *α_j_* and *β_j_* independently over *j* from the mixture distribution $\frac{\pi }{2}\times U(-l-0.2,-l)+\frac{\pi }{2}\times U(l,l+0.2)+(1-\pi )\times \delta_{0},$ where π∈(0,1) and *δ*_0_ denotes a point mass at 0. We vary the following factors in the simulations:

1. Degree of confounding: Let *ρ* determine the strength of association between X and Z, ρ∈{0.1,1,1.5} roughly corresponds to weak (+), medium (++), and strong (+++) confounding, respectively. See Supplementary Section S8 for the role of *ρ* in each simulated model.
2. Signal density: π∈{5%,10%,20%} represents low, medium, and high signal density, respectively.
3. Signal effect: l∈{0.2,0.3,0.4} represents weak, moderate, and strong effect, respectively.

We report the empirical FDR and power averaged over 100 simulation runs for all possible combinations of the three factors.

### 5.2 Competing methods

We compare the finite sample performance of the following seven methods.

1. MS-1DFDR: The 1D procedure based on the *t*-statistics for testing $\alpha_{j}=0$ under the full model (see the detailed descriptions of each data generating model in Supplementary Section S8). The 1D procedure is essentially the same as the 2DFDR+ procedure, except that instead of a 2d rejection region, we are searching for a cutoff along a single dimension, namely that of the conditional statistic. The statistics used in this 1D procedure is the model-based statistic, i.e. the *z*-statistic (or *t*-statistic, depending on the model) corresponding to the coefficient of **X** for a full model fit.
2. RV-1DFDR: The 1D procedure based on the conditional RV coefficient. To account for the potential nonlinearity in the underlying relationship between X and Z (and similarly, Y and Z), the residuals obtained from a cubic spline regression of X on Z (and similarly, Y on Z) have been used in the calculation of the conditional RV coefficient.
3. HSIC-1DFDR: The 1D procedure based on the cHSIC described in Supplementary Section S4.2.2.
4. 2DFDR: The 2DFDR procedure proposed in Yi *et al.* (2021), which is based on linear models with the measurement of the omics feature as the outcome and the covariate of interest and confounders as the predictors.
5. MS-2DFDR+: The proposed 2DFDR+ procedure with $T_{j}^{C}$ and $T_{j}^{M}$ being the *t*-statistics for testing $\alpha_{j}=0$ under the full model and reduced model as described in Supplementary Section S4.1.
6. RV-2DFDR+: The proposed 2DFDR+ procedure with $T_{j}^{C}=\hat{\text{cRV}}(X,Y_{j}|Z)$ and $T_{j}^{M}=\hat{\text{RV}}(X,Y_{j})$ which denote the sample estimates of the conditional and the unconditional RV coefficients, respectively. As before, to account for the potential nonlinearity in the underlying relationship between X and Z (and similarly, Y and Z), the residuals obtained from a cubic spline regression of X on Z (and similarly, Y on Z) have been used in the calculation of the conditional RV coefficient.
7. HSIC-2DFDR+: The proposed 2DFDR+ procedure with $T_{j}^{C}=\hat{\text{cHSIC}}(X,Y_{j}|Z)$ and $T_{j}^{M}=\hat{\text{HSIC}}(X,Y_{j})$, where we set ϵ=0.001 and used the Gaussian kernel with the bandwidth parameter chosen using the median heuristic (Garreau *et al.* 2017).

The 1D procedure can be viewed as a special case of the corresponding 2d procedure by forcing the cutoff of the auxiliary statistic to be zero. As the 2d procedure is searching over a larger rejection region (by allowing the cutoff of the auxiliary statistic to be greater than zero and meanwhile lowering the cutoff for the primary statistic), the proposed 2d procedure is guaranteed to make more rejections in finite sample.

### 5.3 Data generating processes

Throughout the simulations, we set the sample size *n* to be 100, and the number of hypotheses *m* (i.e. the number of features) to be 1000. In the main paper, we consider two cases, remaining cases and detailed models have been discussed in Supplementary Sections S8 and S9.

**Figure 2.**
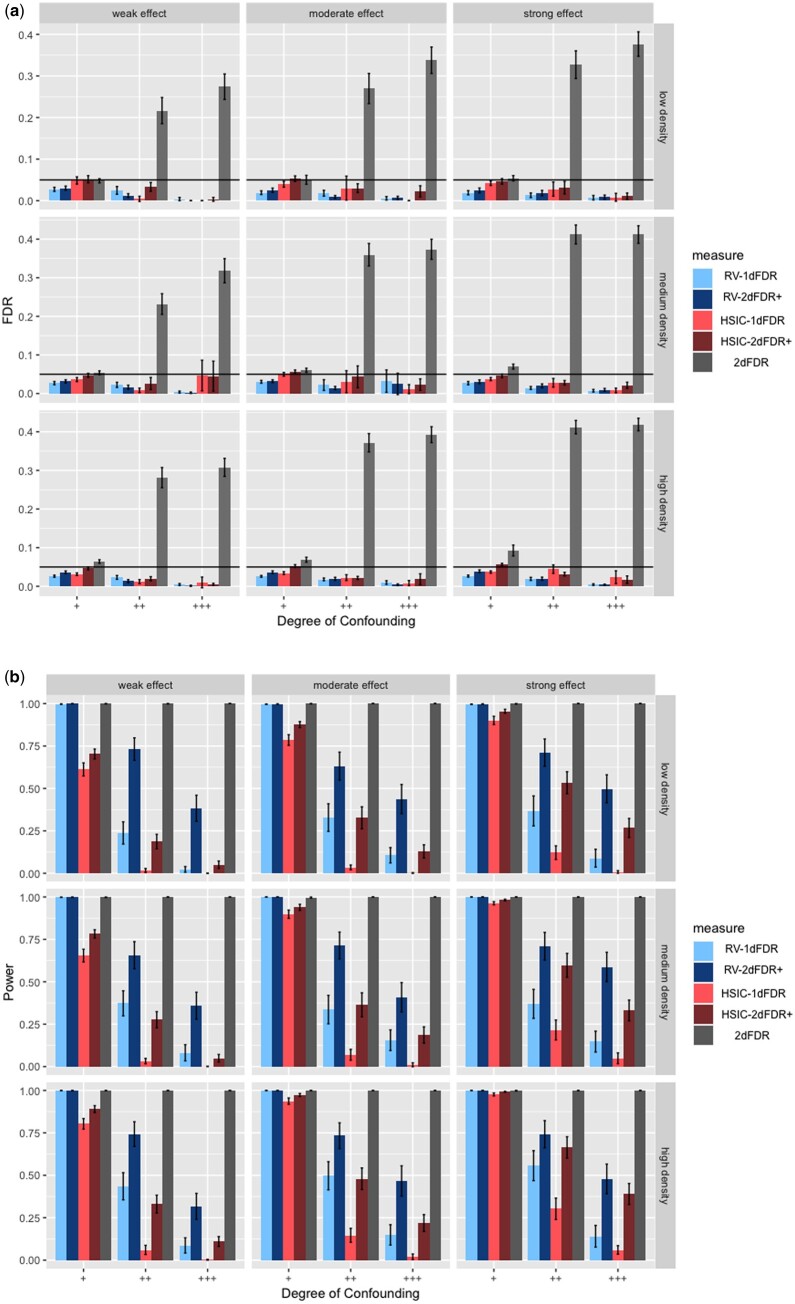
Empirical FDR (a) and power (b) for HSIC-1DFDR, RV-1DFDR, 2DFDR, HSIC-2DFDR+, RV-2DFDR+ for $Y_{j}=\alpha_{j}X^{3}+\beta_{j}e^{Z}+\epsilon_{j}$ and $X∼N(\rho Z^{2},1)$, where Z∼N(0,1). Error bars represent the 95% CIs and the horizontal line in (a) indicates the target FDR level of 0.05.

**Figure 3.**
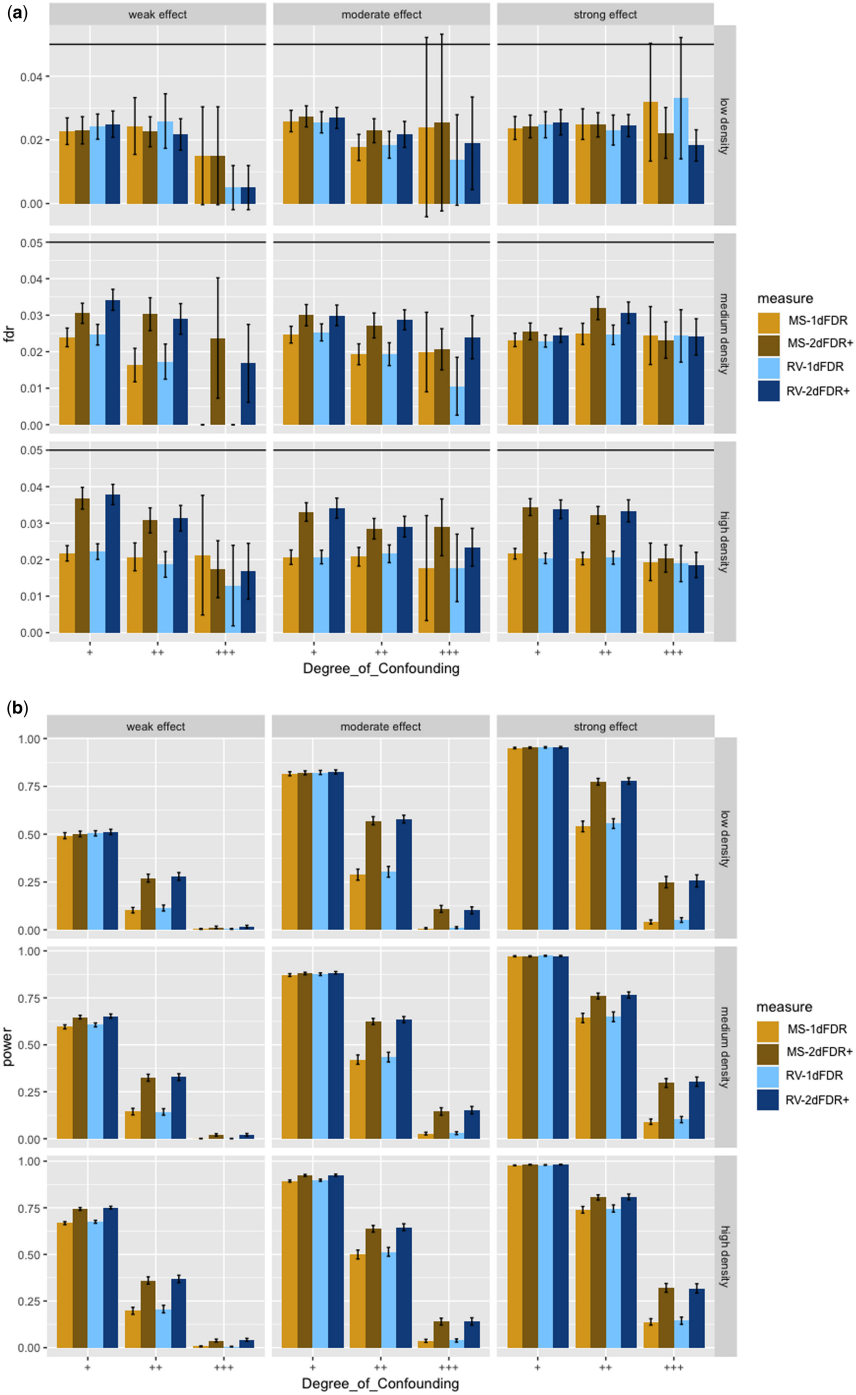
Empirical FDR (a) and power (b) for MS-1DFDR, RV-1DFDR, MS-2DFDR+, RV-2DFDR+ for $Y_{j}∼\text{Bernoulli}({\left(1+e^{-f_{j}(X,Z)}\right)}^{-1})$, where $f_{j}(X,Z)=\alpha_{j}X+\beta_{j}Z,\;X∼N(\rho Z,1)$ and Z∼N(0,1). Error bars represent the 95% CIs. The horizontal line in (a) indicates the target FDR level of 0.05.

- *Linear/Nonlinear models with continuous X and Z.*
(6) $$Y_{j}=\alpha_{j}f(X)+\beta_{j}g(Z)+\epsilon_{j},\;\epsilon_{j}∼N(0,1),\;j=1,\ldots ,m.$$
*X* and *Z* are associated with each other through the following model:
  (7) X∼N(ρh(Z),1), Z∼N(0,1),
  where *ρ* controls the degree of confounding and h:R→R is a possibly nonlinear function. We rescale *X* to dissociate any possible entanglement between signal strength and the degree of confounding. This type of simulation setup has been used in Models 1–4 to explore the effect of the relations among *X*, *Y_j_*, and *Z* on the FDR and power. The empirical FDR and power of RV-1DFDR, HSIC-1DFDR, 2DFDR, RV-2DFDR+, and HSIC-2DFDR+ are summarized in Figs 2 (nonlinear) and 4 (linear) and again in Supplementary Figs S2 and S3. MS-1DFDR and MS-2DFDR+ have not been included under this scenario because the statistics associated with these procedures are directly proportional to the statistics in RV-1DFDR and RV-2DFDR+, respectively.
- *Binary response.* The following logistic regression model has been considered:
  (8) $$Y_{j}∼\text{Bernoulli}(p_{j}),\;\;\text{log}\;\left(\frac{p_{j}}{1-p_{j}}\right)=\alpha_{j}X+\beta_{j}Z,$$
  with $X∼N(\rho Z^{2},1)$ and Z∼N(0,1). We implement the MS-1DFDR, RV-1DFDR, MS-2DFDR+, and RV-2DFDR+, and report the results in Fig. 3. In MS-2DFDR+, $T_{j}^{C}$ is the statistic for testing $\alpha_{j}=0$ under the full model (8) and $T_{j}^{M}$ is the statistic for testing $\alpha_{j}=0$ by forcing $\beta_{j}=0$ in Equation (8).

In Supplementary Section S9, we report some additional numerical results under the following scenarios: (1) Linear/nonlinear models with discrete *X* and continuous *Z*; (2) Linear models with discrete *X* and *Z*; (3) Count response; (4) FWER control; (5) global null; (6) dependent errors, and (7) separating the effects of the densities of the signal of interest and the confounder signal.

### 5.4 Simulation results

**Figure 4.**
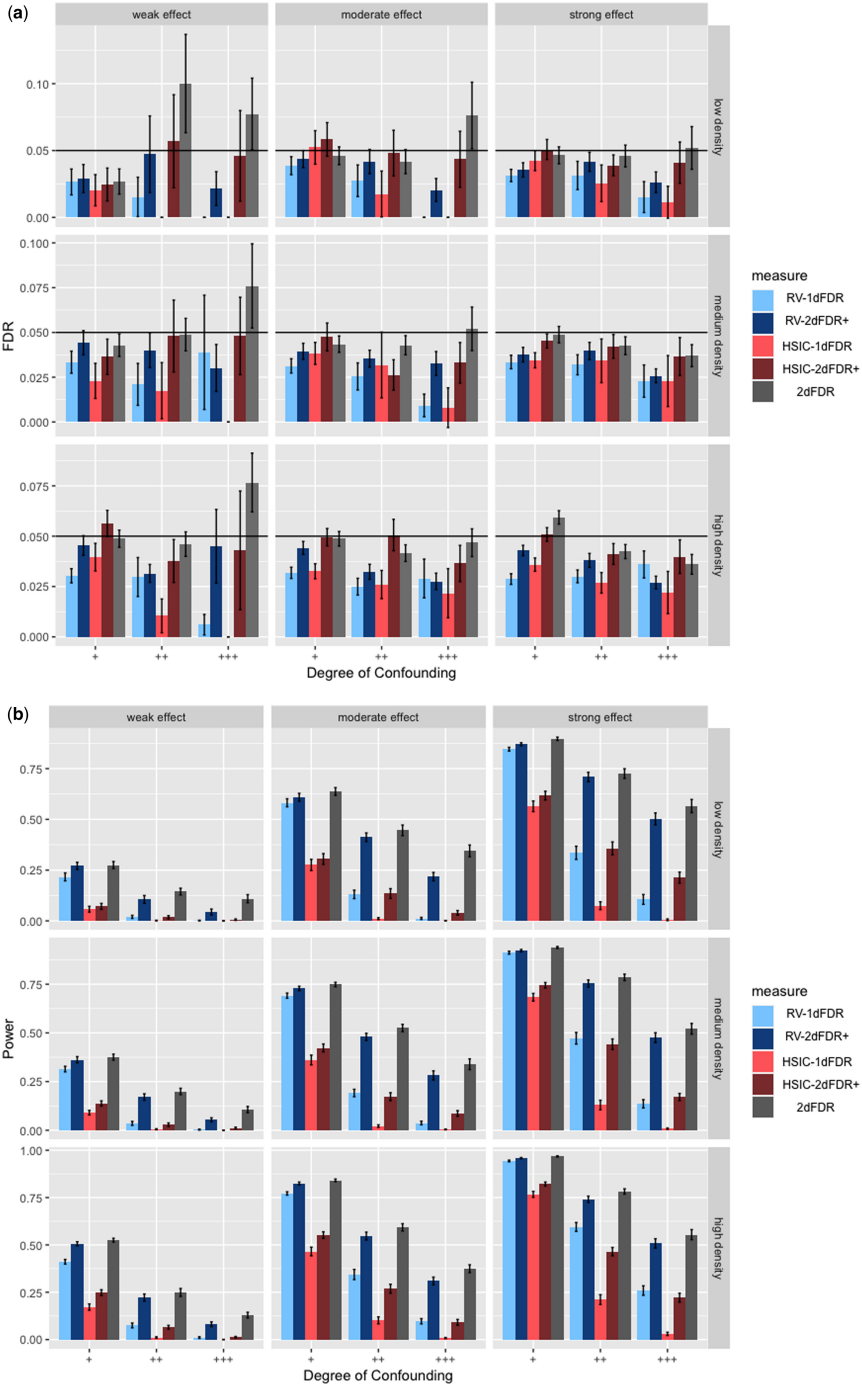
Empirical FDR (a) and power (b) for HSIC-1DFDR, RV-1DFDR, 2DFDR, HSIC-2DFDR+, RV-2DFDR+ under the model $Y_{j}=\alpha_{j}X+\beta_{j}Z+\epsilon_{j}$ and X∼N(ρZ,1), where Z∼N(0,1). Error bars represent the 95% CIs and the horizontal line in (a) indicates the target FDR level of 0.05.

We now discuss the major simulation findings under the scenario described in the previous section. Full simulation results are summarized in Figs 2–4 and Supplementary Figs S2–S16. For continuous *X* and *Z*, when the underlying models between *Y* and (*X*, *Z*), and *X* and *Z* are both linear (see Fig. 4), all the methods provide tight FDR control except for the 2DFDR which has slight FDR inflation in some instances when the confounding effect is strong. In contrast, the proposed RV-2DFDR+, which is equivalent to MS-2DFDR+, controls the FDR at the target level across all cases, indicating more robustness of the proposed method than the original 2DFDR. In terms of power, we observe that the power decreases as the confounding effect becomes stronger for all procedures. The 2d procedure is comparable to the 1D counterpart when the confounding effect is weak but is substantially more powerful when the confounding effect is strong. We also observe that RV-2DFDR+ is comparable to 2DFDR and is more powerful than HSIC-2DFDR+. When the underlying model is nonlinear, 2DFDR suffers from severe FDR inflation (Fig. 2a). In contrast, 2DFDR+ controls the FDR at the target level across different cases. Among the 2DFDR+ variants, RV-2DFDR+ delivers the highest power in most cases. Similar to the linear case, the power decreases (Fig. 2b) as the degree of confounding increases. The 2d procedure is again comparable to its 1D counterpart when the degree of confounding is weak, but the power improvement is more apparent as the degree of confounding increases, especially under low signal density and weak effect, which is often the case in real-world data. For binary outcome (Fig. 3), 2DFDR is not applicable. As before, RV-2DFDR+ and MS-2DFDR+ show substantial improvement in power over their 1D counterparts while keeping the FDR under control. The power decreases as the degree of confounding increases, but the increase in power is higher for lower signal density.

## 6 Real data analysis

### 6.1 Microbiome data

In the first example, we analyze a microbiome dataset in the R package *GUniFrac*. Here, we use the data from the left oropharynx of 32 nonsmokers and 28 smokers (*n *=* *60). The microbiome composition was profiled using 16S rRNA gene-targeted sequencing and processed using the QIIME bioinformatics pipeline (D’Argenio *et al.* 2014), resulting in a count table recording the frequencies of 856 detected OTUs (operational taxonomic units). Sex is a confounding factor in this dataset, with more smokers in males (odds ratio equals 2.3). The aim here is to identify smoking-associated OTUs while adjusting sex.

**Figure 5.**
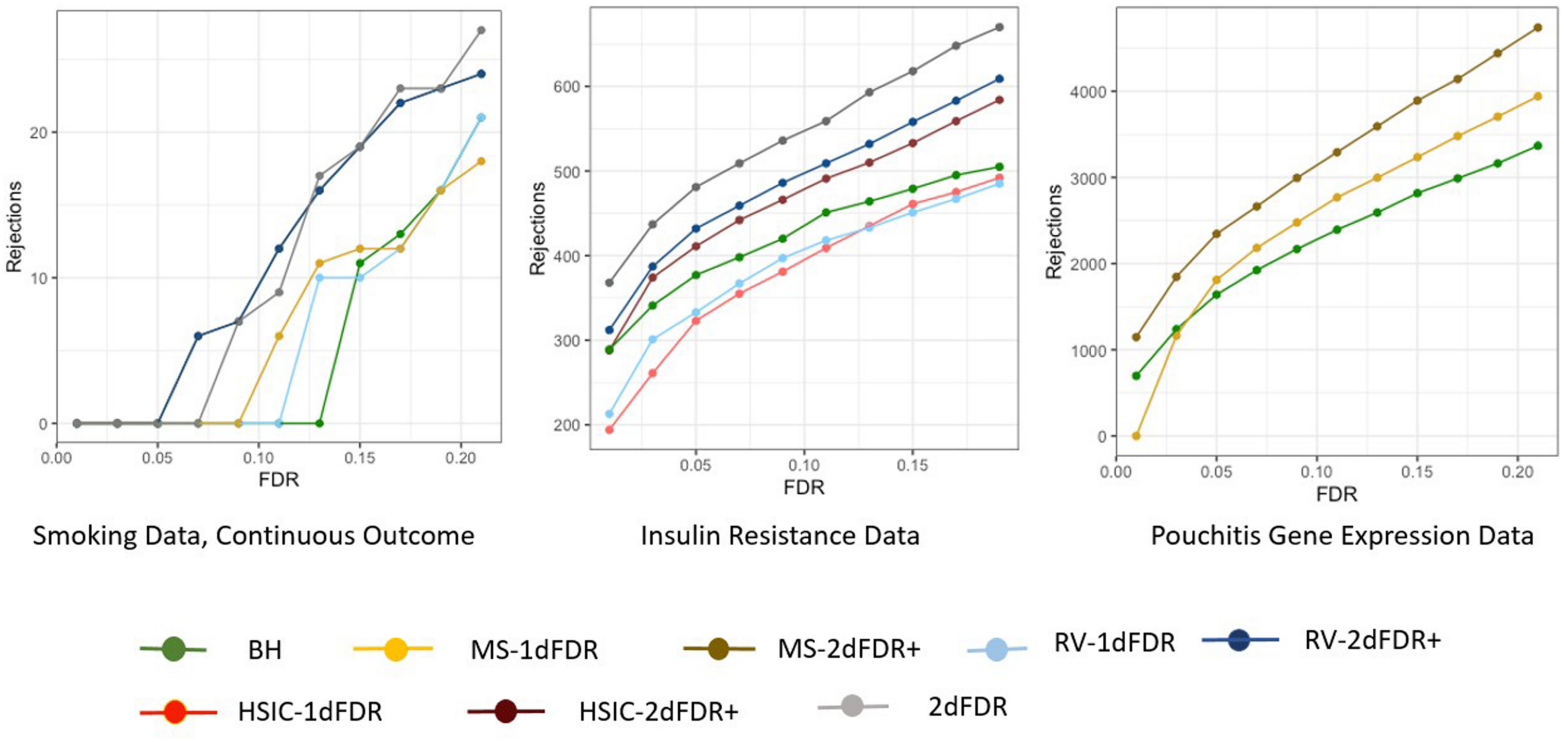
Number of rejections versus FDR for different methods in the smoking (continuous outcomes), insulin resistance, and Pouchitis gene expression dataset.

For illustration purposes, the OTU abundances were treated as both continuous and binary outcomes. The results for the binary outcomes are given in the Supplementary Material. We first filtered out the OTUs occurring in less than 10% of the subjects, which resulted in a total of 174 OTUs. The OTU abundance data were then transformed using a center log-ratio transformation, adding a pseudocount of 0.5. The numbers of rejections for varying levels of FDR (ranging from 0 to 0.2) were calculated for the following methods: Benjamini–Hochberg (BH; Benjamini and Hochberg 1995) procedure, 2DFDR, MS-2DFDR+, RV-2DFDR+, MS-1DFDR, RV-1DFDR. The BH procedure was applied to the *P*-values corresponding to the tests of significance of the coefficients of insulin resistance (IR) in a linear regression model with the IR and body mass index (BMI) being the predictors. The numbers of rejections at different FDR levels are shown in Fig. 5, panel 1. The trend is consistent with the simulations, where we have observed that the 2DFDR+ procedure is more powerful than the corresponding 1DFDR procedure and RV-2DFDR+ makes the highest number of rejections. In addition, we produced a Venn diagram (Supplementary Fig. S17) of the rejected features for each method at the FDR level 0.10 to visualize the degree to which the rejected features in various methods overlap. We find that at the level 0.1, MS-2DFDR+ successfully identifies all the seven features identified by the 2DFDR procedure and five additional features.

### 6.2 Metabolomics data

Next, we consider an Insulin Resistance dataset (Pedersen *et al.* 2018) where the goal is to identify serum metabolites associated with IR while controlling the effect of the BMI of the individual. A group of 289 nondiabetic Danish adults was recruited for the study, where their IR was estimated by homeostatic model assessment (HOMA-IR) (Matthews *et al.* 1985). Untargeted metabolome profiles were generated on fasting serum samples, producing measurements on 325 polar metabolites and 876 molecular lipids (collectively called serum metabolites, *m *=* *1201). The BMI of a subject is a confounding factor as the IR of a subject is largely influenced by the BMI (correlation coefficient =0.57). In this example, 2DFDR discovers the largest number of metabolites (481 at 5% FDR), followed by RV-2DFDR+ (432 metabolites at 5% FDR). Both are a significant improvement over RV-1DFDR (333), HSIC-1DFDR (323), and the BH procedure (377). The comparison of the number of rejections versus FDR level for all methods is displayed in Fig. 5, panel 2.

Again, the result generally agrees with the findings from the simulation studies. While 2DFDR is the most powerful in this example, its inflated type I error rate observed in many nonlinear simulation setups raises some concern about the reliability of the rejections solely found by itself.

Supplementary Figure S18 shows the Venn diagram of the serum metabolites detected by the different methods and their degree of overlap at FDR =0.05 is provided. It is interesting to note that while RV-2DFDR+ and 2DFDR have detected 403 metabolites in common, the BH procedure has significantly fewer overlapping metabolites with either of these methods.

### 6.3 Gene expression data

Finally, we consider a Pouchitis dataset (Morgan *et al.* 2015), where the goal is to identify gene expressions associated with patient outcomes in a cohort with ileal pouch-anal anastomosis (IPAA) surgery in the past one year, adjusting for potential confounders such as antibiotics use and sex. The expression levels of 19 908 genes measured in the J-pouch for *n *=* *74 candidates were considered. The conditioning variables were sex, smoking status, and antibiotic use in the previous month. The variable of interest is the disease outcome, including FAP (Familial Adenomatous Polyposis), No Pouchitis, Acute Pouchitis, Chronic Pouchitis, and Crohn’s Disease like Inflammation. As the variable of interest is nominal, we did not use the RV coefficients in this case. Figure 5, panel 3 shows the number of genes identified as associated with the disease outcome conditioning on sex, smoking status, and antibiotic usage. At the FDR level of 0.05, the 2DFDR+ identifies the maximum number of genes (2345), followed by MS-1DFDR (1811) and BH procedure (1640), respectively.

## 7 Conclusion

We have proposed a general framework (2DFDR+) for performing multiple hypotheses testing while adjusting for confounding effects. Within this new framework, the conditional distribution of the omics features given the variable of interest and confounders can be arbitrary and completely unknown. The framework is flexible by allowing the joint use of any conditional and marginal independence tests, continuous/binary/count/multivariate responses, and various ways of modeling the conditional distribution of the variable of interest given the confounders. As a general methodology, 2DFDR+ can be applied to multiple types of omics data. In view of the numerical results, we recommend using RV-2DFDR+ (based on the spline-transformed variables) under most scenarios due to its robustness and efficiency. In cases where the RV-based statistics are not applicable, for instance, when either of X,Y or Z are categorical, or when Y is discrete (e.g. originating from a Poisson or Negative Binomial distribution), the model-based statistics are recommended. Table 1 summarizes the statistics that we recommend using under different scenarios.

**Table 1.** Recommended statistics under various scenarios.^a^

Y	X	Z	*T^M^* and *T^C^*
C	C	C	RV and cRV
Ct/D	C	C	Model-based statistics (GLM)
Ct/D	Ct	C	Model-based statistics (GLM)
Ct/D	C	Ct	Model-based statistics (GLM)
C	Ct	Ct	Model-based statistics (ANOVA)
C	Ct	C	Model-based statistics (ANCOVA)
Ct	Ct	Ct	$\chi^{2}$ and CMH-statistics

a C, continuous variable; Ct, categorical variable; D, discrete variable.

## Supplementary Material

btad563_Supplementary_Data

## Data Availability

The Smoking Microbiome Data is publicly available in the R package GUniFrac. The remaining datasets and the code is available at https://github.com/asmita112358/tdfdr.np.
