# Supplementary material for: A general framework for powerful confounder adjustment in omics association studies
Source: Bioinformatics. 2023 Sep 9;39(9):btad563. doi: 10.1093/bioinformatics/btad563 (PMC10539716; doi:10.1093/bioinformatics/btad563)
Supplement: btad563_Supplementary_Data [file btad563_supplementary_data.zip › supp.pdf]

The supplement contains more discussions on FDR control of our proposed method, the 2d FWER-controlling procedure, the technical details, asymptotic power analysis, the DGPs used in the simulation studies, and additional numerical results.

S1. FDR Control and Power Analysis

We first show that under the global null, a version of the 2dFDR+ procedure provides finite sample FDR control (or equivalently FWER control). The key to the proof relies on the symmetry of the statistics $\{(T^M_{j,b}, T^C_{j,b}) : j = 1, 2, \ldots, m\}$ across the index b. Let $\{(t_1(s), t_2(s)) \in \mathbb{R}^+ \times \mathbb{R}^+ : 1 \le s \le \mathcal{S}\}$ be a sequence of thresholds such that $t_1(s) \le t_1(s')$ and $t_2(s) \le t_2(s')$ for $1 \le s < s' \le \mathcal{S}$. Let $V^b(s) = \sum_{j=1}^m \mathbf{1}\{T^M_{j,b} \ge t_1(s), T^C_{j,b} \ge t_2(s)\}$ for $0 \le b \le B$. Define

$$s^* = \min\left\{1 \le s \le \mathcal{S} : \frac{(B+1)^{-1}\sum_{b=0}^B V^b(s)}{1 \vee V^0(s)} \le q\right\}.$$

Then we reject any hypothesis such that $T^M_{j,0} \ge t_1(s^*)$ and $T^C_{j,0} \ge t_2(s^*)$.

Theorem ST1. *Under the global null, the above 2dFDR+ procedure provides finite sample FDR control or equivalently FWER control.*

Under general setting, the symmetry among $(T^M_{j,b}, T^C_{j,b})_{j=1}^m$ no longer holds and the finite sample FDR control is not guaranteed. Fortunately, we manage to show that 2dFDR+ provides asymptotic FDR control as n, m both diverge to infinity. To achieve this goal, we impose the following assumptions.

Assumption 1. *Conditional on $(\mathbf{X}, \mathbf{Z})$, Y_j's are independent across $1 \le j \le m$. Moreover, for $j \in \mathcal{M}_0$, Y_j's are independent conditional on $\mathbf{Z}$.*

Assumption 1 requires that the marginal models of Y_j conditional on $\mathbf{X}$ and $\mathbf{Z}$ are independent across $1 \le j \le m$. For instance, consider the model

$$\begin{aligned} Y_j &= u_j(\mathbf{X}) + v_j(\mathbf{Z}) + \epsilon_j, \quad j \notin \mathcal{M}_0, \\ Y_j &= v_j(\mathbf{Z}) + \epsilon_j, \quad j \in \mathcal{M}_0, \end{aligned} \tag{S1}$$

where $u_j(\cdot)$ and $v_j(\cdot)$ are some functions defined on $\mathbb{R}^p$ and $\mathbb{R}^d$ respectively. In this case, Assumption 1 is fulfilled provided that ϵ_j's are independent across j.

Remark 1. In the simulation studies, we also consider the case where Y_j are dependent conditional on $(\mathbf{X}, \mathbf{Z})$. The result shows that our method is indeed robust to such dependence; see Figure S10 of the supplement.

Assumption 2. *Recall that m_0 denotes the number of true null hypotheses. Suppose $m_0/m \to \pi_0 \in (0, 1)$ and there exist two continuous bivariate functions $\widetilde{V}(\cdot,\cdot)$ and $\widetilde{S}(\cdot,\cdot)$ defined on $\mathbb{R}^+ \times \mathbb{R}^+$ such that*

$$\left|\frac{1}{m_0}\sum_{j\in\mathcal{M}_0} \mathbb{P}(T^M_j \ge t_1, T^C_j \ge t_2 | \widetilde{\mathbf{X}}, \widetilde{\mathbf{Z}}) - \widetilde{V}(t_1, t_2)\right| \to^p 0,$$

$$\left|\frac{1}{m}\sum_{j=1}^m \mathbb{P}(T^M_j \ge t_1, T^C_j \ge t_2 | \widetilde{\mathbf{X}}, \widetilde{\mathbf{Z}}) - \widetilde{S}(t_1, t_2)\right| \to^p 0,$$

for any fixed $t_1, t_2 \ge 0$.

Assumption 2 is a high-level condition. We justify this assumption under model (S1) in Section S2. Our next assumption is similar to the requirement in Theorem 4 of [10], which ensures the existence of cut-off values to control the FDR at level q. It reduces the search region for the optimal cut-offs to a rectangle of the form $[0, t_{0,1}] \times [0, t_{0,2}]$.

Assumption 3. *Assume that there exist $t_{0,1}$ and $t_{0,2}$ such that,*

$$\frac{\pi_0 \widetilde{V}(t_{0,1}, 0) + u_1}{\widetilde{S}(t_{0,1}, 0)} \le q' < q, \qquad \frac{\pi_0 \widetilde{V}(0, t_{0,2}) + u_2}{\widetilde{S}(0, t_{0,2})} \le q'' < q,$$

and $\widetilde{S}(t_{0,1}, t_{0,2}) > c > 0$, where π_0 is defined in Assumption 2, $u_1 = \limsup m^{-1}\sum_{j\in\mathcal{M}_1} \mathbb{P}_0(T^M_j \ge t_{0,1}|\widetilde{\mathbf{Y}}_j, \widetilde{\mathbf{Z}})$ and $u_2 = \limsup m^{-1}\sum_{j\in\mathcal{M}_1} \mathbb{P}_0(T^C_j \ge t_{0,2}|\widetilde{\mathbf{Y}}_j, \widetilde{\mathbf{Z}})$ with $\mathcal{M}_1 = \{1 \le j \le p : H_{0,j}$ is non-null$\}$.

Recall that in practice, $\mathbb{P}_{\mathbf{X}|\mathbf{Z}}$ is often unknown and has to be estimated from the data. Under suitable assumptions on the estimated conditional distribution, the FDR can still be controlled at the target level. Specifically, suppose

$$\mathbf{X}_{i,b} \sim^{\text{ind}} \widehat{\mathbb{P}}_{\mathbf{X}|\mathbf{Z}}(\cdot|\mathbf{Z}_i), \quad i = 1, 2, \ldots, n, \quad b = 1, 2, \ldots, B,$$

for some $\widehat{\mathbb{P}}_{\mathbf{X}|\mathbf{Z}}$ estimated from the data. Additionally, we define

$$\begin{aligned} &\widehat{\mathbb{P}}(T^M_j \ge t_1, T^C_j \ge t_2 | \widetilde{\mathbf{Y}}_j, \widetilde{\mathbf{Z}}) \\ &= \int \mathbf{1}\{T^M_j(\widetilde{\mathbf{x}}, \widetilde{\mathbf{Y}}_j) \ge t_1, T^C_j(\widetilde{\mathbf{x}}, \widetilde{\mathbf{Y}}_j, \widetilde{\mathbf{Z}}) \ge t_2\} d\prod_{i=1}^n \widehat{\mathbb{P}}_{\mathbf{X}|\mathbf{Z}}(\mathbf{x}_i|\mathbf{Z}_i), \end{aligned}$$

where $\widehat{\mathbb{P}}(\cdot|\widetilde{\mathbf{Y}}_j, \widetilde{\mathbf{Z}})$ can be viewed as an estimate of $\mathbb{P}_0(\cdot|\widetilde{\mathbf{Y}}_j, \widetilde{\mathbf{Z}})$ based on $\mathbf{X}_{i,b} \sim^{\text{ind}} \widehat{\mathbb{P}}_{\mathbf{X}|\mathbf{Z}}$ for $1 \le i \le n$ and $1 \le b \le B$. Define

$$\hat{Q}_{n,m}(t_1, t_2) = \frac{1}{m_0}\sum_{j\in\mathcal{M}_0} \widehat{\mathbb{P}}(T^M_j \ge t_1, T^C_j \ge t_2 | \widetilde{\mathbf{Y}}_j, \widetilde{\mathbf{Z}})$$

We make the following two assumptions on $\widehat{\mathbb{P}}_{\mathbf{X}|\mathbf{Z}}$.

Assumption 4. *As $m, n, B \to \infty$,*

$$P\left(\left(\int \mathbf{1}\{T^M_j(\widetilde{\mathbf{x}}, \widetilde{\mathbf{Y}}_j) \ge t_1, T^C_j(\widetilde{\mathbf{x}}, \widetilde{\mathbf{Y}}_j, \widetilde{\mathbf{Z}}) \ge t_2\} \left(d\mathbb{P}(\widetilde{\mathbf{x}}|\widetilde{\mathbf{Z}}) - d\widehat{\mathbb{P}}(\widetilde{\mathbf{x}}|\widetilde{\mathbf{Z}})\right)\right) \le 0, \forall 1 \le j \le m\right) \to 1.$$

A sufficient condition for Assumption 4 is that

$$\max_i d^2_{\mathrm{H}}(\widehat{\mathbb{P}}_{\mathbf{X}|\mathbf{Z}}(\mathbf{x}_i|\mathbf{Z}_i), \mathbb{P}_{\mathbf{X}|\mathbf{Z}}(\mathbf{x}_i|\mathbf{Z}_i)) = o_p(n^{-1/2})$$

with d_{H} denoting the Hellinger distance, see Remark 4 in the supplement for the derivations. The same condition has been considered previously in [2].

Assumption 5. *Assume that there exist $\hat{t}_{0,1}$ and $\hat{t}_{0,2}$ such that*

$$\frac{\pi_0\widehat{Q}(\hat{t}_{0,1},0)+v_1}{\widetilde{S}(\hat{t}_{0,1},0)} \le q' < q, \quad \frac{\pi_0\widehat{Q}(0,\hat{t}_{0,2})+v_2}{\widetilde{S}(0,\hat{t}_{0,2})} \le q'' < q \quad a.s$$

and $\widetilde{S}(\hat{t}_{0,1},\hat{t}_{0,2})) > c_2 > 0$ *a.s, where*

$$v_1 = \limsup m^{-1}\sum_{j\in\mathcal{M}_1}\widehat{\mathbb{P}}(T_j^M \ge \hat{t}_{0,1}|\widetilde{\mathbf{Y}}_j,\widetilde{\mathbf{Z}})$$

and

$$v_2 = \limsup m^{-1}\sum_{j\in\mathcal{M}_1}\widehat{\mathbb{P}}(T_j^C \ge \hat{t}_{0,2}|\widetilde{\mathbf{Y}}_j,\widetilde{\mathbf{Z}})$$

a.s.

Assumption 5 is similar to Assumption 3, but in terms of the estimated conditional distribution $\widehat{\mathbb{P}}_{\mathbf{X}|\mathbf{Z}}$.

We now turn to the power analysis of the oracle 2dFDR+ procedure. We argue that 2dFDR+ is, in general, more powerful than the corresponding 1d procedure based on the conditional independence statistics alone. Assume without loss of generality that T_j^M takes non-negative values. The intuition is that for $t_1 = 0$, the first dimension does not screen out any null hypothesis and only the second dimension is effective in identifying signals. In this case, 2dFDR+ reduces to the corresponding 1d procedure, where we reject $H_{0,j}$ if $T_j^C \ge t^*$ with t^* being the solution to the following problem

$$\max_t \sum_{j=1}^m \left\{T_j^C \ge t\right\} \quad \text{subject to} \quad \frac{\sum_{j=1}^m \bar{F}_{j,B}(0,t)}{1\vee\sum_{j=1}^m \mathbf{1}\{T_j^C\ge t\}} \le q.$$

Clearly, $(0,t^*)$ is in the feasible set $\mathcal{F}_q$ of the optimization problem in Section 3.2. Therefore, we have $\sum_{j=1}^m \mathbf{1}\{T_j^M \ge t_1^*, T_j^C \ge t_2^*\} \ge \sum_{j=1}^m\{T_j^C \ge t^*\}$. In other words, 2dFDR+ is guaranteed to deliver at least as many rejections as the corresponding 1d procedure does.

Define $\mathrm{FP}_{2\mathrm{d}}$ and $\mathrm{FP}_{1\mathrm{d}}$ as the number of false positives for 2dFDR+ and the associated 1d procedure respectively. Similarly, we let $\mathrm{TP}_{2\mathrm{d}}$ and $\mathrm{TP}_{1\mathrm{d}}$ be the number of true positives. Suppose Assumptions 1-3 hold and both procedures make rejections (i.e., $\mathrm{FP}+\mathrm{TP}>0$). In addition, assume

$$\frac{\mathrm{FP}_{1\mathrm{d}}}{\mathrm{FP}_{1\mathrm{d}}+\mathrm{TP}_{1\mathrm{d}}} = q_1, \quad \frac{\mathrm{FP}_{2\mathrm{d}}}{\mathrm{FP}_{2\mathrm{d}}+\mathrm{TP}_{2\mathrm{d}}} = q_2, \tag{S2}$$

for some $0 \le q_1, q_2 \le 1$. As 2dFDR+ makes more rejections, i.e., $\mathrm{FP}_{1\mathrm{d}}+\mathrm{TP}_{1\mathrm{d}} \le \mathrm{FP}_{2\mathrm{d}}+\mathrm{TP}_{2\mathrm{d}}$, we must have

$$\mathrm{TP}_{2\mathrm{d}} \ge \frac{1-q_2}{1-q_1}\mathrm{TP}_{1\mathrm{d}}.$$

When $q_2 \le q_1$, $\mathrm{TP}_{2\mathrm{d}} \ge \mathrm{TP}_{1\mathrm{d}}$, i.e., 2dFDR+ makes more true rejections. In general, we have the following lower bound on the number of true positives for 2dFDR+.

Corollary SC1. *Under Assumptions 1-3 and as $B \to +\infty$, we have for any $\epsilon > 0$,*

$$\mathbb{P}(TP_{2d} \ge (1-q-\epsilon)TP_{1d}) \to 1. \tag{S3}$$

As ϵ can be arbitrarily small, (S3) suggests that with the FDR controlled at level q, 2dFDR+ asymptotically achieves at least $100(1-q)\%$ true rejections of the 1d procedure in the worst-case scenario. For instance, with $q = 5\%$, the power loss compared to the 1d procedure is at most 5%. We refer the readers to Section S7 of the supplement for more discussions on the asymptotic power of 2dFDR+.

Remark 2. Since 2dFDR+ depends on the marginal independence statistic to filter features, when the confounder and variable of interest have opposite effects on the feature with similar magnitude, they will cancel out each other's effect, and the feature could be excluded erroneously in the first dimension. The optimal cutoff of T_j^M is thus determined based on the tradeoff between power reduction due to erroneously excluding these relevant features in the first dimension and power increase due to reducing the multiple testing burden and increasing the signal density in the second dimension. If the true signals can only be revealed after adjusting for the confounder, for example, when the true and confounding signals co-locate with opposite directions, the marginal independence test statistics will not be informative. In this case, the best cutoff on T_j^M should be 0 and 2dFDR+ is then reduced to the 1d procedure. In finite samples, it may not always be possible to reduce 2dFDR+ to the 1d procedure exactly. Nevertheless, as argued above, the power loss is relatively moderate even in the worst-case scenario.

S2. Discussions on Assumption 2

We provide further discussions on Assumption 2 and justify it under model (S1) with

$$u_j(\mathbf{X}) = \sum_{k=1}^{J_1}\alpha_{k,j}B_{k,\mathbf{X}}(\mathbf{X}), \quad v_j(\mathbf{Z}) = \sum_{k=1}^{J_2}\beta_{k,j}B_{k,\mathbf{Z}}(\mathbf{Z}),$$
$$\boldsymbol{\epsilon}_j = (\epsilon_{1,j},\dots,\epsilon_{n,j})^\top \sim N(0,\sigma_j^2\mathbf{I}),$$

where $B_{k,\mathbf{X}}(\cdot):\mathbb{R}^p\to\mathbb{R}$ and $B_{k,\mathbf{Z}}(\cdot):\mathbb{R}^d\to\mathbb{R}$ are some known basis functions. Define $\mathbf{B}_\mathbf{X} = (B_{k,\mathbf{X}}(\mathbf{X}_i))_{1\le i\le n,1\le k\le J_1} \in \mathbb{R}^{n\times J_1}$ and $\mathbf{B}_\mathbf{Z} = (B_{k,\mathbf{Z}}(\mathbf{Z}_i))_{1\le i\le n,1\le k\le J_2} \in \mathbb{R}^{n\times J_2}$. Let $\mathbf{P}_\mathbf{Z}^\perp$ be the orthogonal projection onto the column space of $\mathbf{B}_\mathbf{Z}$. We consider the statistics

$$T_j^M = \hat{\sigma}_j^{-2}\|(\mathbf{B}_\mathbf{X}^\top\mathbf{B}_\mathbf{X})^{-1/2}\mathbf{B}_\mathbf{X}^\top\widetilde{\mathbf{Y}}_j\|^2,$$
$$T_j^C = \hat{\sigma}_j^{-2}\|(\mathbf{B}_\mathbf{X}^\top\mathbf{P}_\mathbf{Z}^\perp\mathbf{B}_\mathbf{X})^{-1/2}\mathbf{B}_\mathbf{X}^\top\mathbf{P}_\mathbf{Z}^\perp\widetilde{\mathbf{Y}}_j\|^2,$$

where $\hat{\sigma}_j^2$ is a consistent variance estimator of σ_j^2 such that $\hat{\sigma}_j^2 \to^p \sigma_j^2$. Conditional on $(\widetilde{\mathbf{X}},\widetilde{\mathbf{Z}})$, $(\mathbf{B}_\mathbf{X}^\top\mathbf{B}_\mathbf{X})^{-1/2}\mathbf{B}_\mathbf{X}^\top\widetilde{\mathbf{Y}}_j$ and $(\mathbf{B}_\mathbf{X}^\top\mathbf{P}_\mathbf{Z}^\perp\mathbf{B}_\mathbf{X})^{-1/2}\mathbf{B}_\mathbf{X}\mathbf{P}_\mathbf{Z}^\perp\widetilde{\mathbf{Y}}_j$ jointly follow the multivariate normal distribution with the mean

$$\begin{pmatrix}(\mathbf{B}_\mathbf{X}^\top\mathbf{B}_\mathbf{X})^{1/2}\boldsymbol{\alpha}_j + (\mathbf{B}_\mathbf{X}^\top\mathbf{B}_\mathbf{X})^{-1/2}(\mathbf{B}_\mathbf{X}^\top\mathbf{B}_\mathbf{Z})\boldsymbol{\beta}_j \\ (\mathbf{B}_\mathbf{X}^\top\mathbf{P}_\mathbf{Z}^\perp\mathbf{B}_\mathbf{X})^{1/2}\boldsymbol{\alpha}_j\end{pmatrix}$$

and the covariance matrix

$$\sigma_j^2\begin{pmatrix}\mathbf{I} & (\mathbf{B}_\mathbf{X}^\top\mathbf{B}_\mathbf{X})^{-1/2}(\mathbf{B}_\mathbf{X}^\top\mathbf{P}_\mathbf{Z}^\perp\mathbf{B}_\mathbf{X})^{1/2} \\ (\mathbf{B}_\mathbf{X}^\top\mathbf{P}_\mathbf{Z}^\perp\mathbf{B}_\mathbf{X})^{1/2}(\mathbf{B}_\mathbf{X}^\top\mathbf{B}_\mathbf{X})^{-1/2} & \mathbf{I}\end{pmatrix}.$$

Define

$$\boldsymbol{\Sigma}_\mathbf{X} = \mathrm{cov}(\widetilde{\mathbf{B}}_\mathbf{X}),$$
$$\boldsymbol{\Sigma}_{\mathbf{X}\mathbf{Z}} = \mathrm{cov}(\widetilde{\mathbf{B}}_\mathbf{X},\widetilde{\mathbf{B}}_\mathbf{Z})$$

and

$$\boldsymbol{\Sigma}_{\mathbf{X}|\mathbf{Z}} = \boldsymbol{\Sigma}_\mathbf{X} - \boldsymbol{\Sigma}_{\mathbf{X}\mathbf{Z}}\boldsymbol{\Sigma}_\mathbf{Z}^{-1}\boldsymbol{\Sigma}_{\mathbf{Z}\mathbf{X}},$$

where

$$\widetilde{\mathbf{B}}_\mathbf{X} = (B_{1,\mathbf{X}}(\mathbf{X}),\dots,B_{J_1,\mathbf{X}}(\mathbf{X}))^\top$$

and

$$\widetilde{\mathbf{B}}_{\mathbf{Z}} = (B_{1,\mathbf{Z}}(\mathbf{Z}), \ldots, B_{J_2,\mathbf{X}}(\mathbf{Z}))^\top.$$

By the law of large numbers, we have

$$n^{-1}\mathbf{B}_{\mathbf{X}}^\top \mathbf{B}_{\mathbf{X}} \to^p \mathbf{\Sigma}_{\mathbf{X}},$$
$$n^{-1/2}(\mathbf{B}_{\mathbf{X}}^\top \mathbf{B}_{\mathbf{X}})^{-1/2}(\mathbf{B}_{\mathbf{X}}^\top \mathbf{B}_{\mathbf{Z}}) \to^p \mathbf{\Sigma}_{\mathbf{X}}^{-1/2}\mathbf{\Sigma}_{\mathbf{XZ}},$$
$$n^{-1}\mathbf{B}_{\mathbf{X}}^\top \mathbf{P}_{\mathbf{Z}}^\perp \mathbf{B}_{\mathbf{X}} \to^p \mathbf{\Sigma}_{\mathbf{X}|\mathbf{Z}}.$$

In this case, we have

$$\widetilde{V}(t_1,t_2) = \lim_{m,n\to+\infty} \frac{1}{m_0} \sum_{j\in\mathcal{M}_0} F(t_1,t_2;0,\sqrt{n}\boldsymbol{\beta}_j/\sigma_j),$$

$$\widetilde{S}(t_1,t_2) = \lim_{m,n\to+\infty} \frac{1}{m} \sum_{j=1}^{m} F(t_1,t_2;\sqrt{n}\boldsymbol{\alpha}_j/\sigma_j,\sqrt{n}\boldsymbol{\beta}_j/\sigma_j),$$

with $F(t_1,t_2;\mathbf{a},\mathbf{b}) = \mathbb{P}(\|\mathbf{V}_{1,j}\|^2 > t_1, \|\mathbf{V}_{2,j}\|^2 > t_2)$, where $(\mathbf{V}_{1,j},\mathbf{V}_{2,j})$ follow the multivariate normal distribution with the mean

$$\begin{pmatrix} \mathbf{\Sigma}_{\mathbf{X}}^{1/2}\mathbf{a} + \mathbf{\Sigma}_{\mathbf{X}}^{-1/2}\mathbf{\Sigma}_{\mathbf{XZ}}\mathbf{b} \\ \mathbf{\Sigma}_{\mathbf{X}|\mathbf{Z}}^{1/2}\mathbf{a} \end{pmatrix}$$

and the covariance matrix

$$\begin{pmatrix} \mathbf{I} & \mathbf{\Sigma}_{\mathbf{X}}^{-1/2}\mathbf{\Sigma}_{\mathbf{X}|\mathbf{Z}}^{1/2} \\ \mathbf{\Sigma}_{\mathbf{X}|\mathbf{Z}}^{1/2}\mathbf{\Sigma}_{\mathbf{X}}^{-1/2} & \mathbf{I} \end{pmatrix}.$$

If $(\sqrt{n}\boldsymbol{\alpha}_j/\sigma_j, \sqrt{n}\boldsymbol{\beta}_j/\sigma_j)$ follows some distribution $\mathcal{F}$ independently across j and conditional on $\boldsymbol{\alpha}_j = 0$, $\sqrt{n}\boldsymbol{\beta}_j/\sigma_j$ follows the distribution $\mathcal{F}_0$ independently for $j \in \mathcal{M}_0$, then we have

$$\widetilde{V}(t_1,t_2) = \int F(t_1,t_2,(0,\boldsymbol{\beta}))d\mathcal{F}_0(\boldsymbol{\beta}),$$
$$\widetilde{S}(t_1,t_2) = \int F(t_1,t_2,(\boldsymbol{\alpha},\boldsymbol{\beta}))d\mathcal{F}(\boldsymbol{\alpha},\boldsymbol{\beta}).$$

S3. Estimating the Conditional Distributions

As the conditional distribution $\mathbb{P}_{\mathbf{X}|\mathbf{Z}}$ is seldom known, we need to estimate it from the data. There are indeed several ways of generating samples from $\mathbb{P}_{\mathbf{X}|\mathbf{Z}}$. Examples include classical methods such as residual permutation [12] and parametric bootstrap [4] as well as modern approaches such as conditional generative adversarial network (conditional GAN) [7, 15]. In the following subsections, we shall describe the residual permutation, residual bootstrap, and parametric bootstrap in more detail. Compared to the conditional GAN, these procedures are more suitable for omics applications, given the limited sample sizes in many omics association studies.

S3.1. Residual permutation and residual bootstrap

When $\mathbf{X}$ is a continuous random vector, we can model the relationship between $\widetilde{\mathbf{X}} \in \mathbb{R}^{n\times p}$ and $\widetilde{\mathbf{Z}} \in \mathbb{R}^{n\times d}$ through a multivariate linear regression model given by

$$\widetilde{\mathbf{X}} = \widetilde{\mathbf{Z}}\mathbf{B} + \mathbf{E}, \tag{S4}$$

where $\mathbf{B} \in \mathbb{R}^{d\times p}$ is the matrix of coefficients and $\mathbf{E} \in \mathbb{R}^{n\times p}$ is the error matrix. Consider the following strategy to generate samples from $\mathbb{P}_{\mathbf{X}|\mathbf{Z}}$.

Step 1: Fitting regression model. Fit the multivariate linear regression model in (S4). Let $\widehat{\mathbf{E}} = \widetilde{\mathbf{X}} - \widetilde{\mathbf{Z}}\widehat{\mathbf{B}}$ be the residuals from the fitted model, $\widehat{\mathbf{B}}$ being the least squares estimate of $\mathbf{B}$.

Step 2: Residual permutation. Permute the rows of the residual matrix $\widehat{\mathbf{E}}$ and denote the resulting matrix by $\widehat{\mathbf{E}}^*$. Let $\widetilde{\mathbf{X}}_b = (\mathbf{X}_{1,b},\ldots,\mathbf{X}_{n,b})^\top = \widetilde{\mathbf{Z}}\widehat{\mathbf{B}} + \widehat{\mathbf{E}}^*$.

Step 2′: Residual bootstrap. Let $\widehat{\mathbf{E}}^{**}$ be a $n \times p$ matrix whose rows are sampled with replacement from those of $\widehat{\mathbf{E}}$. Let $\widetilde{\mathbf{X}}_b = (\mathbf{X}_{1,b},\ldots,\mathbf{X}_{n,b})^\top = \widetilde{\mathbf{Z}}\widehat{\mathbf{B}} + \widehat{\mathbf{E}}^{**}$.

Remark 3. To allow nonlinearity, we can replace Z_i by $(g_1(Z_i),\ldots,g_{d'}(Z_i)) \in \mathbb{R}^{d'}$ for some transformations $(g_1,\ldots,g_{d'})$ in the multivariate regression model.

S3.2. Parametric bootstrap

Suppose the conditional distribution of $\mathbf{X}$ given $\mathbf{Z}$ takes the parametric form of $\mathbb{P}_{\mathbf{X}|\mathbf{Z}}(\mathbf{X}_i|\mathbf{Z}_i;\theta)$, where $\theta \in \Theta \subseteq \mathbb{R}^r$ is an unknown parameter. It is natural to estimate the parameter by maximizing the conditional log-likelihood

$$\widehat{\theta} = \arg\max_{\theta\in\Theta} \sum_{i=1}^{n} \log \mathbb{P}_{\mathbf{X}|\mathbf{Z}}(\mathbf{X}_i|\mathbf{Z}_i;\theta).$$

Then we can generate $\mathbf{X}_{i,b}$ from the estimated likelihood $\mathbb{P}_{\mathbf{X}|\mathbf{Z}}(\mathbf{X}_i|\mathbf{Z}_i;\widehat{\theta})$. For example, suppose $\mathbf{X}$ is a Bernoulli random variable with the conditional success probability given by $\{1+\exp(-\mathbf{Z}_i^\top\theta)\}^{-1}$. Then we can sample $\mathbf{X}_{i,b}$ from the Bernoulli distribution with success probability $\{1+\exp(-\mathbf{Z}_i^\top\widehat{\theta})\}^{-1}$, where $\widehat{\theta}$ is an estimate of θ by fitting a logistic model to the data with $\widetilde{\mathbf{X}}$ being the binary response and $\widetilde{\mathbf{Z}}$ being the covariates.

S4. Independence Tests

We review some parametric and nonparametric unconditional/conditional independence tests and discuss their use within our framework. In Section S4.1, we focus on the model-based (parametric) independence tests. In Sections S4.2.1-S4.2.2, we consider two types of nonparametric independence tests targeting linear and nonlinear dependence respectively. These three types of independence tests will all be implemented in our numerical studies.

S4.1. Model-based statistics

Suppose the conditional likelihood of Y_j given $\mathbf{X}$ and $\mathbf{Z}$ has the form of

$$\mathbb{P}_{Y_j|\mathbf{X},\mathbf{Z}}(Y_j|\mathbf{X}^\top\boldsymbol{\alpha}_j + \mathbf{Z}^\top\boldsymbol{\beta}_j). \tag{S5}$$

The log-likelihood function based on the observations is given by

$$L_{n,j}(\boldsymbol{\alpha}_j,\boldsymbol{\beta}_j) = \sum_{i=1}^{n} \log \mathbb{P}_{Y_j|\mathbf{X},\mathbf{Z}}(Y_{i,j}|\mathbf{X}_i^\top\boldsymbol{\alpha}_j + \mathbf{Z}_i^\top\boldsymbol{\beta}_j).$$

In this case, testing $H_{0,j}$ is equivalent to testing whether $\boldsymbol{\alpha}_j$ is zero. Thus we let T_j^C be a statistic for testing $\boldsymbol{\alpha}_j = 0$ under the model (S5). Examples include the Wald test and the likelihood-ratio test. To test the marginal independence, we consider the reduced model $\mathbb{P}_{Y_j|\mathbf{X},\mathbf{Z}}(Y_j|\mathbf{X}^\top\boldsymbol{\alpha}_j)$ by forcing $\boldsymbol{\beta}_j = 0$ in (S5). Under the reduced model, we let T_j^M be a statistic for testing $\boldsymbol{\alpha}_j = 0$, which can be viewed as testing the marginal independence $Y_j \perp\!\!\!\perp \mathbf{X}$. When $\mathbb{P}_{Y_j|\mathbf{X},\mathbf{Z}}$ is the likelihood

function associated with a linear model with Gaussian error, we can let T_j^C and T_j^M be the adjusted and unadjusted z-statistics considered in [13]. In this sense, the statistics in [13] fall into our framework.

S4.2. Nonparametric dependence metrics

Nonparametric dependence testing, aiming to determine whether two random vectors are dependent without specifying the exact parametric forms of the distributions, is one of the fundamental problems in statistics. Classical metrics or test statistics for dependence testing include the RV coefficient, rank correlation coefficient, and nonparametric Cramŕr-von Mises type statistics. Modern approaches are built on distance and kernel embedding, which can detect non-linear and non-monotone dependence. Notable examples include the distance covariance [11], Hilbert-Schmidt independence criterion (HSIC) [5, 6] and the sign distance covariance [1]. Below we shall review the RV coefficient and HSIC and discuss their conditional versions for testing the conditional independence.

S4.2.1. RV coefficients

Pearson correlation and partial correlation coefficients are perhaps the most commonly used nonparametric dependence metrics for measuring marginal and conditional dependence. Here we describe the RV coefficient and its conditional version as multivariate generalizations of the squared Pearson correlation coefficient and the squared partial correlation coefficient for detecting linear and conditional linear dependence.

For two random vectors $\mathbf{U}$ and $\mathbf{V}$, we let $\Sigma_{\mathbf{U},\mathbf{V}}$ be the covariance matrix between $\mathbf{U}$ and $\mathbf{V}$. The RV coefficient between $\mathbf{X}$ and Y_j is defined as

$$\mathrm{RV}(\mathbf{X}, Y_j) = \frac{\mathrm{tr}(\boldsymbol{\Sigma}_{\mathbf{X},Y_j}\boldsymbol{\Sigma}_{Y_j,\mathbf{X}})}{\sqrt{\mathrm{tr}(\Sigma^2_{\mathbf{X},\mathbf{X}})\mathrm{tr}(\Sigma^2_{Y_j,Y_j})}}.$$

To estimate the RV coefficient, we simply replace the covariance matrices in the definition above with the sample covariance matrices.

To introduce the conditional version of the RV coefficient, we let $\mathbf{e}_{\mathbf{X}}$ and e_{Y_j} be the residuals by regressing $\mathbf{X}$ and Y_j on $\mathbf{Z}$ respectively. The conditional RV coefficient is defined as

$$\mathrm{cRV}(\mathbf{X}, Y_j|\mathbf{Z}) = \mathrm{cRV}(\mathbf{e}_{\mathbf{X}}, e_{Y_j}).$$

Similar to Remark 3, to account for the nonlinear dependence of $\mathbf{X}$ and Y_j on $\mathbf{Z}$, we can replace $\mathbf{Z}$ by certain basis function transform on it, e.g., spline transformation.

S4.2.2. Hilbert-Schmidt independence criterion

Hilbert-Schmidt Independence Criterion (HSIC) was introduced as a kernel-based independence measure by [5, 6]. Let $k_p(\cdot,\cdot)$ be a reproducing kernel Hilbert space (RKHS) kernel defined on $\mathbb{R}^p \times \mathbb{R}^p$. Commonly used kernels in this context include the Gaussian kernel and the Laplacian kernel. The HSIC for quantifying the strength of dependence between $\mathbf{X}$ and Y_j can be defined as

$$\begin{aligned}\mathrm{HSIC}(\mathbf{X}, Y_j) =& \mathbb{E}[k_p(\mathbf{X}, \mathbf{X}')k_1(Y_j, Y_j')] \\ &+ \mathbb{E}[k_p(\mathbf{X}, \mathbf{X}')]\mathbb{E}[k_1(Y_j, Y_j')] \\ &- 2\mathbb{E}[k_p(\mathbf{X}, \mathbf{X}')k_1(Y_j, Y_j'')]\end{aligned}$$

where $(\mathbf{X}', Y_j')$ and $(\mathbf{X}'', Y_j'')$ are independent copies of $(\mathbf{X}, Y_j)$. When k_p and k_1 are characteristic kernels [9], HSIC completely characterizes the dependence in the sense that $\mathbf{X}$ and Y_j are independent if and only if $\mathrm{HSIC}(\mathbf{X}, Y_j) = 0$. To estimate the HSIC, define $\mathbf{K}_{\mathbf{X}} = (k_{\mathbf{X},ab})_{a,b=1}^n$ with $k_{\mathbf{X},ab} = k_p(\mathbf{X}_a, \mathbf{X}_b)$ and $\mathbf{K}_{Y_j} = (k_{Y_j,ab})_{a,b=1}^n$ with $k_{Y_j,ab} = k_1(Y_{a,j}, Y_{b,j})$. Let $\mathbf{H} = \mathbf{I} - n^{-1}\mathbf{1}\mathbf{1}^\top$ with $\mathbf{1}$ being the n-dimensional vector of all ones. Set $\widetilde{\mathbf{K}}_{\mathbf{X}} = \mathbf{H}\mathbf{K}_{\mathbf{X}}\mathbf{H}$ and $\widetilde{\mathbf{K}}_{Y_j} = \mathbf{H}\mathbf{K}_{Y_j}\mathbf{H}$. The sample HSIC is defined as

$$\widehat{\mathrm{HSIC}}(\mathbf{X}, Y_j) = \frac{1}{n}\mathrm{Tr}(\widetilde{\mathbf{K}}_{\mathbf{X}}\widetilde{\mathbf{K}}_{Y_j}),$$

which has been shown to be a consistent estimator, see [5].

A conditional version of the HSIC (cHSIC) for measuring and testing conditional dependence was proposed in [14]. Here we describe the construction of their statistic. Let $\mathbf{K}_{\mathbf{X},\mathbf{Z}} = (k_{\mathbf{X},\mathbf{Z},ab})_{a,b=1}^n$ with $k_{\mathbf{X},\mathbf{Z},ab} = k_{p+d}((\mathbf{X}_a, \mathbf{Z}_a), (\mathbf{X}_b, \mathbf{Z}_b))$ and define $\mathbf{K}_{Y_j,\mathbf{Z}}$ in a similar way. Denote by $\widetilde{\mathbf{K}}_{\mathbf{X},\mathbf{Z}} = \mathbf{H}\mathbf{K}_{\mathbf{X},\mathbf{Z}}\mathbf{H}$ and $\widetilde{\mathbf{K}}_{Y_j,\mathbf{Z}} = \mathbf{H}\mathbf{K}_{Y_j,\mathbf{Z}}\mathbf{H}$ the centered versions of $\mathbf{K}_{\mathbf{X},\mathbf{Z}}$ and $\mathbf{K}_{Y_j,\mathbf{Z}}$ respectively. Further, define $\widetilde{\mathbf{K}}_{\mathbf{X}\mathbf{Z}|\mathbf{Z}} = \epsilon^2(\widetilde{\mathbf{K}}_{\mathbf{X}\mathbf{Z}} + \epsilon\mathbf{I})^{-1}\widetilde{\mathbf{K}}_{\mathbf{X}\mathbf{Z}}(\widetilde{\mathbf{K}}_{\mathbf{X}\mathbf{Z}} + \epsilon\mathbf{I})^{-1}$ and $\widetilde{\mathbf{K}}_{Y_j\mathbf{Z}|\mathbf{Z}} = \epsilon^2(\widetilde{\mathbf{K}}_{Y_j\mathbf{Z}} + \epsilon\mathbf{I})^{-1}\widetilde{\mathbf{K}}_{Y_j\mathbf{Z}}(\widetilde{\mathbf{K}}_{Y_j\mathbf{Z}} + \epsilon\mathbf{I})^{-1}$ for some small positive constant ϵ. The sample cHSIC is given by

$$\widehat{\mathrm{cHSIC}}(\mathbf{X}, Y_j|\mathbf{Z}) = \frac{1}{n}\mathrm{Tr}(\widetilde{\mathbf{K}}_{\mathbf{X}\mathbf{Z}|\mathbf{Z}}\widetilde{\mathbf{K}}_{Y_j\mathbf{Z}|\mathbf{Z}}).$$

We refer the readers to [14] for more detailed properties about the cHSIC.

S5. Family-wise error rate control

Family-wise error rate (FWER), referring to the probability of making one false discovery, provides more stringent type I error rate control. It is preferable to the FDR if the overall conclusion from various individual inferences is likely to be erroneous when at least one of them is, or the existence of a single false claim would cause significant loss. It is natural to ask whether our method can be modified to control other error measures such as FWER. Here we describe such a procedure to control the FWER. Given the rejection rule $\mathbf{1}\{T_j^M \geq t_1, T_j^C \geq t_2\}$, we let $\widetilde{\mathrm{FWER}}(t_1, t_2) := \sum_{j=1}^m \bar{F}_{j,B}(t_1, t_2)$ be an estimate of the FWER. We choose the optimal cut-off value as the one that maximizes the number of rejections while controls the FWER estimate at a prespecified level q:

$$(\check{t}_1, \check{t}_2) = \operatorname*{arg\,max}_{(t_1,t_2)\in\mathcal{G}_q} \sum_{j=1}^m \mathbf{1}\{T_j^M \geq t_1, T_j^C \geq t_2\},$$

where $\mathcal{G}_q = \{(t_1, t_2) \in \mathbb{R}^+ \times \mathbb{R}^+ : \widetilde{\mathrm{FWER}}(t_1, t_2) \leq q\}$. Then we reject $H_{0,j}$ whenever $T_j^M \geq \check{t}_1$ and $T_j^C \geq \check{t}_2$. We name the above procedure 2dFWER+. In Section S9, we investigate its finite sample performance and report the empirical FWER and power for 2dFWER+ and its corresponding 1d version (1dFWER) in Figures S15 and S16.

S6. Details of FDR control

In this section, we prove the main theoretical results in the paper. We first present the following lemma which was recently proved in [8].

Lemma 1 (Dvoretzky–Kiefer–Wolfowitz inequality). *Let $\boldsymbol{\xi}_1, \cdots, \boldsymbol{\xi}_n$ be independent d-dimensional random vectors with the distribution function $F(\mathbf{t}) = \mathbb{P}(\boldsymbol{\xi}_i \leq \mathbf{t})$, where $\boldsymbol{\xi}_i \leq \mathbf{t}$ means that $\xi_{ij} \leq t_j$ for $\boldsymbol{\xi}_i = (\xi_{i1}, \ldots, \xi_{id})$ and $1 \leq j \leq$*

d. Denote the standard empirical distribution function by $F_n(\mathbf{t}) = n^{-1}\sum_{i=1}^{n} \mathbf{1}\{\boldsymbol{\xi}_i \le \mathbf{t}\}$. *Then we have*

$$\mathbb{P}\left(\sup_{\mathbf{t}\in\mathbb{R}^d} |F_n(\mathbf{t}) - F(\mathbf{t})| > \epsilon\right) \le d(n+1)\exp\left(-2n\epsilon^2\right).$$

We now present proof of the main theoretical results.

Proof of Theorem ST1 Define the filtration

$$\mathcal{F}_s = \sigma\left(\left\{\mathbf{1}\{T_{j,b}^M \ge t_1(a)\}, \mathbf{1}\{T_{j,b}^C \ge t_2(a)\}\right\}_{1\le j\le m, 0\le b\le B} : 1 \le a \le s\right)$$

for $1 \le s \le \mathcal{S}$ and the process $U(s) = \widetilde{V}^0(s)/\{\sum_{b=0}^{B} \widetilde{V}^b(s)\}$, which is adapted to the filtration $\mathcal{F}_s$. The conditional distribution of $\widetilde{V}^b(t)$ given the sigma-field $\sigma(\{\mathbf{1}\{T_{j,b}^M \ge t_1(a)\}, \mathbf{1}\{T_{j,b}^M \ge t_2(a)\}\}_{1\le j\le m} : 1 \le a \le s)$ with $s < t$ are the same across all $b = 0, 1, \ldots, B$. By the symmetry, we must have for $s < t$, $\mathbb{E}[U(t)|\mathcal{F}_s] = (B+1)^{-1}$. Thus $U(t) - 1/(B+1)$ is a martingale difference sequence. Also, we have $\{s^* \le t\} \in \mathcal{F}_t$. Therefore, s^* is a stopping time. By the optional stopping time theorem,

$$\mathbb{E}[U(s^*)] = \frac{1}{B+1}. \tag{S6}$$

Recall from the definition of s^* that

$$\frac{(B+1)^{-1}\sum_{b=0}^{B} V^b(s^*)}{1 \vee V^0(s^*)} \le q. \tag{S7}$$

Using (S6) and (S7), we obtain

$$\mathbb{E}\left[\frac{\widetilde{V}^0(s^*)}{1 \vee V^0(s^*)}\right] \le (B+1)q\mathbb{E}\left[\frac{\widetilde{V}^0(s^*)}{\sum_{b=0}^{B} V^b(s^*)}\right] = (B+1)q\mathbb{E}\left[U(s^*)\right] = q.$$

□

Proof of Theorem 1. For $(t_1, t_2) \in \mathbb{R}^+ \times \mathbb{R}^+$, define the following processes

$$S_{n,m}(t_1,t_2) = m^{-1}\sum_{j=1}^{m} \mathbf{1}\{T_j^M \ge t_1, T_j^C \ge t_2\},$$

$$V_{n,m}(t_1,t_2) = m_0^{-1}\sum_{j\in\mathcal{M}_0} \mathbf{1}\{T_j^M \ge t_1, T_j^C \ge t_2\},$$

$$Q_{n,m}(t_1,t_2) = m_0^{-1}\sum_{j\in\mathcal{M}_0} \mathbb{P}_0(T_j^M \ge t_1, T_j^C \ge t_2|\widetilde{\mathbf{Y}}_j, \widetilde{\mathbf{Z}}).$$

We divide the proof into two steps. In Step 1, we obtain some uniform convergence results while in Step 2, we apply these results to show the FDR control.

Step 1. Conditional on $(\widetilde{\mathbf{X}}, \widetilde{\mathbf{Z}})$, $\mathbf{1}\{T_j^M \ge t_1, T_j^C \ge t_2\}$ are independent across $j \in \mathcal{M}_0$. By Lemma 1, we have

$$\sup_{t_1\le t_{0,1}, t_2\le t_{0,2}} \left|\frac{1}{m_0}\sum_{j\in\mathcal{M}_0}\left[\mathbf{1}\{T_j^M \ge t_1, T_j^C \ge t_2\} - \mathbb{P}(T_j^M \ge t_1, T_j^C \ge t_2|\widetilde{X}, \widetilde{\mathbf{Z}})\right]\right| \to^p 0. \tag{S8}$$

By Assumption 1, conditional on $\widetilde{\mathbf{Z}}$ and for any fixed t_1 and t_2, $\mathbb{P}(T_j^M \ge t_1, T_j^C \ge t_2|\widetilde{\mathbf{Y}}_j, \widetilde{\mathbf{Z}})$ are independent across $j \in \mathcal{M}_0$. Therefore, by the law of large numbers,

$$\frac{1}{m_0}\sum_{j\in\mathcal{M}_0}\left\{\mathbb{P}(T_j^M \ge t_1, T_j^C \ge t_2|\widetilde{\mathbf{Y}}_j, \widetilde{\mathbf{Z}}) - \mathbb{P}(T_j^M \ge t_1, T_j^C \ge t_2|\widetilde{\mathbf{Z}})\right\} \to^p 0.$$

Following the proof of the Glivenko-Cantelli Theorem, we can strengthen the point-wise convergence to the uniform convergence, i.e.,

$$\sup_{t_1\le t_{0,1}, t_2\le t_{0,2}} \left|\frac{1}{m_0}\sum_{j\in\mathcal{M}_0}\left\{\mathbb{P}(T_j^M \ge t_1, T_j^C \ge t_2|\widetilde{\mathbf{Y}}_j, \widetilde{\mathbf{Z}}) - \mathbb{P}(T_j^M \ge t_1, T_j^C \ge t_2|\widetilde{\mathbf{Z}})\right\}\right| \to^p 0. \tag{S9}$$

Similarly, the result in Assumption 2 can also be strengthened to the uniform convergence, i.e.,

$$\sup_{t_1\le t_{0,1}, t_2\le t_{0,2}} \left|\frac{1}{m_0}\sum_{j\in\mathcal{M}_0} \mathbb{P}(T_j^M \ge t_1, T_j^C \ge t_2|\widetilde{X}, \widetilde{\mathbf{Z}}) - \widetilde{V}(t_1,t_2)\right| \to^p 0. \tag{S10}$$

It implies that

$$\begin{aligned}
&\sup_{t_1\le t_{0,1}, t_2\le t_{0,2}} \left|\frac{1}{m_0}\sum_{j\in\mathcal{M}_0} \mathbb{P}(T_j^M \ge t_1, T_j^C \ge t_2|\widetilde{\mathbf{Z}}) - \widetilde{V}(t_1,t_2)\right| \\
=&\sup_{t_1\le t_{0,1}, t_2\le t_{0,2}} \left|\mathbb{E}\left[\frac{1}{m_0}\sum_{j\in\mathcal{M}_0} \mathbb{P}(T_j^M \ge t_1, T_j^C \ge t_2|\widetilde{\mathbf{X}}, \widetilde{\mathbf{Z}}) - \widetilde{V}(t_1,t_2)\middle|\widetilde{\mathbf{Z}}\right]\right| \\
\le&\mathbb{E}\left[\sup_{t_1\le t_{0,1}, t_2\le t_{0,2}} \left|\frac{1}{m_0}\sum_{j\in\mathcal{M}_0} \mathbb{P}(T_j^M \ge t_1, T_j^C \ge t_2|\widetilde{\mathbf{Z}}) - \widetilde{V}(t_1,t_2)\right|\middle|\widetilde{\mathbf{Z}}\right] \to^p 0,
\end{aligned} \tag{S11}$$

by Lebesgue's dominated convergence theorem. Combining (S8), (S9), (S10) and (S11) together, we get

$$\sup_{t_1\le t_{0,1}, t_2\le t_{0,2}} \left|V_{n,m}(t_1,t_2) - \widetilde{V}(t_1,t_2)\right| \to^p 0, \tag{S12}$$

$$\sup_{t_1\le t_{0,1}, t_2\le t_{0,2}} \left|Q_{n,m}(t_1,t_2) - \widetilde{V}(t_1,t_2)\right| \to^p 0. \tag{S13}$$

Using similar arguments by conditioning on $(\widetilde{\mathbf{X}}, \widetilde{\mathbf{Z}})$, we can show that

$$\sup_{t_1\le t_{0,1}, t_2\le t_{0,2}} \left|S_{n,m}(t_1,t_2) - \widetilde{S}(t_1,t_2)\right| \to^p 0. \tag{S14}$$

Following the arguments in the proof of Lemma 8.2 of Cao et al. (2020), we have under Assumptions 1-3 that

$$\begin{aligned}
&\sup_{t_1\le t_{0,1}, t_2\le t_{0,2}} \left|\mathrm{FDP}(t_1,t_2) - \frac{\pi_0\widetilde{V}(t_1,t_2)}{\widetilde{S}(t_1,t_2)}\right| \to^p 0, \\
&\sup_{t_1\le t_{0,1}, t_2\le t_{0,2}} \left|\frac{m_0 Q_{n,m}(t_1,t_2)}{1 \vee mS_{n,m}(t_1,t_2)} - \frac{\pi_0\widetilde{V}(t_1,t_2)}{\widetilde{S}(t_1,t_2)}\right| \to^p 0.
\end{aligned} \tag{S15}$$

Moreover, under the null, we have $\mathbb{E}[\mathbf{1}\{T_{j,b}^M \ge t_1, T_{j,b}^C \ge t_2\}|\widetilde{\mathbf{Y}}_j, \widetilde{\mathbf{Z}}] = \mathbb{P}(T_j^M \ge t_1, T_j^C \ge t_2|\widetilde{\mathbf{Y}}_j, \widetilde{\mathbf{Z}})$ by the way we generate $\mathbf{X}_{i,b}$. Thus $m_0^{-1}\sum_{j\in\mathcal{M}_0}\mathbb{E}[\bar{F}_{j,B}(t_1,t_2) -$

$Q_{n,m}(t_1,t_2)|\widetilde{\mathbf{Z}},\widetilde{\mathbf{Y}}_j, j\in\mathcal{M}_0] = 0$ and

$$\begin{aligned}&\operatorname{var}\left(\frac{1}{m_0}\sum_{j\in\mathcal{M}_0}\{\bar{F}_{j,B}(t_1,t_2)-Q_{n,m}(t_1,t_2)\}\middle|\widetilde{\mathbf{Z}},\widetilde{\mathbf{Y}}_j, j\in\mathcal{M}_0\right)\\ =&\frac{1}{B+1}\operatorname{var}\left(\frac{1}{m_0}\sum_{j\in\mathcal{M}_0}\Big(\mathbf{1}\{T^M_{j,1}\ge t_1, T^C_{j,1}\ge t_2\}\right.\\ &\left.-Q_{n,m}(t_1,t_2)\Big)\middle|\widetilde{\mathbf{Z}},\widetilde{\mathbf{Y}}_j, j\in\mathcal{M}_0\right)\\ \le&\frac{1}{4(B+1)},\end{aligned}$$

where we have used the fact that $\operatorname{var}(X)\le 1/4$ for $X\in[0,1]$. Therefore,

$$\frac{1}{m_0}\sum_{j\in\mathcal{M}_0}\{\bar{F}_{j,B}(t_1,t_2)-Q_{n,m}(t_1,t_2)\}\to^p 0,$$

which can be strengthened to the uniform convergence

$$\sup_{t_1\le t_{0,1},t_2\le t_{0,2}}\left|\frac{1}{m_0}\sum_{j\in\mathcal{M}_0}\{\bar{F}_{j,B}(t_1,t_2)-Q_{n,m}(t_1,t_2)\}\right|\to^p 0.$$

Together with (S15), we obtain

$$\sup_{t_1\le t_{0,1},t_2\le t_{0,2}}\left|\frac{\sum_{j\in\mathcal{M}_0}\bar{F}_{j,B}(t_1,t_2)}{1\vee mS_{n,m}(t_1,t_2)}-\frac{\pi_0\widetilde{V}(t_1,t_2)}{\widetilde{S}(t_1,t_2)}\right|\to^p 0. \tag{S16}$$

In view of Assumption 3, (S16) implies that

$$\begin{aligned}&\mathbb{P}\left(\widetilde{\mathrm{FDP}}(t_{0,1},0)<q,\widetilde{\mathrm{FDP}}(0,t_{0,2})<q\right)\\ =&\mathbb{P}\left(\frac{\sum_{j\in\mathcal{M}_0}\bar{F}_{j,B}(t_{0,1},0)+m_1U_{n,m}(t_{0,1},0)}{1\vee mS_{n,m}(t_{0,1},0)}<q,\right.\\ &\left.\frac{\sum_{j\in\mathcal{M}_0}\bar{F}_{j,B}(0,t_{0,2})+m_1U_{n,m}(0,t_{0,2})}{1\vee mS_{n,m}(0,t_{0,2})}<q\right)\to 1,\end{aligned}$$

where $U_{n,m}(t_1,t_2) = m_1^{-1}\sum_{j\in\mathcal{M}_1}\mathbb{P}_0(T^M_j\ge t_1,T^C_j\ge t_2|\widetilde{\mathbf{Y}}_j,\widetilde{\mathbf{Z}})$. Thus we must have

$$\mathbb{P}(t_1^*\le t_{0,1},t_2^*\le t_{0,2})\to 1.$$

Step 2. Note that $\widetilde{\mathrm{FDP}}(t_1,t_2)\ge\sum_{j\in\mathcal{M}_0}\bar{F}_{j,B}(t_1,t_2)/\{1\vee mS_{n,m}(t_1,t_2)\}$. On the event $t_1^*\le t_{0,1}$ and $t_2^*\le t_{0,2}$ which has probability converging to one, we have

$$\begin{aligned}&\mathrm{FDP}(t_1^*,t_2^*)-\widetilde{\mathrm{FDP}}(t_1^*,t_2^*)\\ \le&\mathrm{FDP}(t_1^*,t_2^*)-\frac{\sum_{j\in\mathcal{M}_0}\bar{F}_{j,B}(t_1^*,t_2^*)}{1\vee mS_{n,m}(t_1^*,t_2^*)}\\ =&\mathrm{FDP}(t_1^*,t_2^*)-\frac{\pi_0\widetilde{V}(t_1^*,t_2^*)}{\widetilde{S}(t_1^*,t_2^*)}+\frac{\pi_0\widetilde{V}(t_1^*,t_2^*)}{\widetilde{S}(t_1^*,t_2^*)}-\frac{\sum_{j\in\mathcal{M}_0}\bar{F}_{j,B}(t_1^*,t_2^*)}{1\vee mS_{n,m}(t_1^*,t_2^*)}\\ \le&\sup_{t_1\le t_{0,1},t_2\le t_{0,2}}\left|\mathrm{FDP}(t_1,t_2)-\frac{\pi_0\widetilde{V}(t_1,t_2)}{\widetilde{S}(t_1,t_2)}\right|\\ &+\sup_{t_1\le t_{0,1},t_2\le t_{0,2}}\left|\frac{\sum_{j\in\mathcal{M}_0}\bar{F}_{j,B}(t_1,t_2)}{1\vee mS_{n,m}(t_1,t_2)}-\frac{\pi_0\widetilde{V}(t_1,t_2)}{\widetilde{S}(t_1,t_2)}\right|=o_p(1).\end{aligned}$$

Thus we have

$$\mathrm{FDP}(t_1^*,t_2^*)\le\widetilde{\mathrm{FDP}}(t_1^*,t_2^*)+o_p(1)=q+o_p(1). \tag{S17}$$

By Lemma 8.3 of [3], we get

$$\limsup_{n,m\to+\infty}\mathbb{E}\left[\mathrm{FDP}(t_1^*,t_2^*)\right]\le q.$$

□

Proof of Corollary 1 Recall that

$$\begin{aligned}&\widehat{\mathbb{P}}(T^M_j\ge t_1,T^C_j\ge t_2|\widetilde{\mathbf{Y}}_j,\widetilde{\mathbf{Z}})\\ =&\int\mathbf{1}\{T^M_j(\widetilde{\mathbf{x}},\widetilde{\mathbf{Y}}_j)\ge t_1,T^C_j(\widetilde{\mathbf{x}},\widetilde{\mathbf{Y}}_j,\widetilde{\mathbf{Z}})\ge t_2\}d\prod_{i=1}^n\widehat{\mathbb{P}}_{\mathbf{X}|\mathbf{Z}}(\mathbf{x}_i|\mathbf{Z}_i),\end{aligned}$$

where $\widehat{\mathbb{P}}(\cdot|\widetilde{\mathbf{Y}}_j,\widetilde{\mathbf{Z}})$ is the estimate of $\mathbb{P}_0(\cdot|\widetilde{\mathbf{Y}}_j,\widetilde{\mathbf{Z}})$ based on $\mathbf{X}_{i,b}\sim^{\mathrm{ind}}\widehat{\mathbb{P}}_{\mathbf{X}|\mathbf{Z}}$. Define

$$\widehat{Q}_{n,m}(t_1,t_2)=\frac{1}{m_0}\sum_{j\in\mathcal{M}_0}\widehat{\mathbb{P}}(T^M_j\ge t_1,T^C_j\ge t_2|\widetilde{\mathbf{Y}}_j,\widetilde{\mathbf{Z}})$$

Following the arguments in the proof of Theorem ??, we can show that

$$\sup_{t_1\le\hat{t}_{0,1},t_2\le\hat{t}_{0,2}}\left|\frac{1}{m_0}\sum_{j\in\mathcal{M}_0}\widehat{F}_{j,B}(t_1,t_2)-\widehat{Q}_{n,m}(t_1,t_2)\right|\to^p 0. \tag{S18}$$

For $j\in\mathcal{M}_1$, note that

$$E\left(\frac{1}{m_1}\sum_{j\in\mathcal{M}_1}\widehat{F}_{j,B}(t_1,t_2)-\frac{1}{m_1}\sum_{j\in\mathcal{M}_1}\widehat{\mathbb{P}}(T^M_j\ge t_1,T^C_j\ge t_2|\widetilde{\mathbf{Y}}_j,\widetilde{\mathbf{Z}})\right)=0$$

and

$$\begin{aligned}&V\left(\frac{1}{m_1}\sum_{j\in\mathcal{M}_1}\widehat{F}_{j,B}(t_1,t_2)-\frac{1}{m_1}\sum_{j\in\mathcal{M}_1}\widehat{\mathbb{P}}(T^M_j\ge t_1,T^C_j\ge t_2|\widetilde{\mathbf{Y}}_j,\widetilde{\mathbf{Z}})\right)\\ =&\frac{1}{m_1^2(B+1)}\sum_{j,j'\in\mathcal{M}_1}\operatorname{cov}(K_j(t_1,t_2),K'_j(t_1,t_2))\\ \le&\frac{1}{B+1}\end{aligned}$$

where $K_j(t_1,t_2)=\mathbf{1}\{T^M_{j,1}\ge t_1,T^C_{j,1}\ge t_2\}-\widehat{\mathbb{P}}(T^M_j\ge t_1,T^C_j\ge t_2|\widetilde{\mathbf{Y}}_j,\widetilde{\mathbf{Z}})$. We can strengthen the result to the uniform convergence, i.e.,

$$\begin{aligned}\sup_{t_1\le\hat{t}_{0,1},t_2\le\hat{t}_{0,2}}&\left|\frac{1}{m_1}\sum_{j\in\mathcal{M}_1}\Big(\widehat{F}_{j,B}(t_1,t_2)-\right.\\ &\left.\widehat{\mathbb{P}}(T^M_j\ge t_1,T^C_j\ge t_2|\widetilde{\mathbf{Y}}_j,\widetilde{\mathbf{Z}})\Big)\right|\to^p 0.\end{aligned} \tag{S19}$$

Using Assumption 4, for any $\epsilon>0$, we have

$$\begin{aligned}&P(Q_{n,m}(t_1,t_2)-\widehat{Q}_{n,m}(t_1,t_2)\le\epsilon)\\ \ge&P\left(\sup_{j\in\mathcal{M}_0}\left(\int\mathbf{1}\{T^M_j(\widetilde{\mathbf{x}},\widetilde{\mathbf{Y}}_j)\ge t_1,T^C_j(\widetilde{\mathbf{x}},\widetilde{\mathbf{Y}}_j,\widetilde{\mathbf{Z}})\ge t_2\}\right.\right.\\ &\left.\left.\left(d\mathbb{P}(\widetilde{\mathbf{x}}|\widetilde{\mathbf{Z}})-d\widehat{\mathbb{P}}(\widetilde{\mathbf{x}}|\widetilde{\mathbf{Z}})\right)\right)\le\epsilon\right)\\ \to&1.\end{aligned}$$

By (S13), for any given (t_1,t_2), we have

$$P(\widetilde{V}(t_1,t_2)-\widehat{Q}_{n,m}(t_1,t_2)\le\epsilon)\to 1.$$

The above statement can be strengthened to the uniform convergence, i.e.,

$$P\left(\sup_{t_1\le \hat t_{0,1}, t_2\le \hat t_{0,2}} \left(\widetilde V(t_1,t_2)-\widehat Q_{n,m}(t_1,t_2)\right)\le \epsilon\right)\to 1.$$

To show this, note that $\widetilde V(t,t')$ is a non-increasing function of t and t'. Given $\epsilon>0$, by the continuity of $\widetilde V$, there exists a partition $t_{1,0}:=0<t_{1,1}<t_{1,2}<\cdots<t_{1,\nu_1}<t_{1,\nu_1+1}:=1$ such that $\widetilde V(t_{1,j},0)-\widetilde V(t_{1,j+1},0)<\epsilon/4$ for all $j=0\ldots\nu_1$. Similarly, given $\epsilon>0$ there exists a partition $t_{2,0}:=0<t_{2,1}<t_{2,2}<\cdots<t_{2,\nu_2}<t_{2,\nu_2+1}:=1$ such that $\widetilde V(0,t_{2,k})-\widetilde V(0,t_{2,k+1})<\epsilon/4$ for all $k=0\ldots\nu_2$. Let $\mathcal G_{\nu_1,\nu_2}:=\{(t_{1,j},t_{2,k}):0\le j\le \nu_1+1, 0\le k\le \nu_2+1\}$. We have

$$P\left(\sup_{(t_1,t_2)\in\mathcal G_{\nu_1,\nu_2}} \left(\widetilde V(t_1,t_2)-\widehat Q_{n,m}(t_1,t_2)\right)\le \frac{\epsilon}{2}\right)\to 1.$$

Then for any (t_1,t_2), we can find j,k such that $t_1\in[t_{1,j},t_{1,j+1}]$ and $t_2\in[t_{2,k},t_{2,k+1}]$. For such a (t_1,t_2)

$$\begin{aligned}\widehat Q_{n,m}(t_1,t_2) &\ge \widehat Q_{n,m}(t_{1,j+1},t_{2,k+1}) \quad (\text{As } \widehat Q_{n,m} \text{ is non-increasing})\\ &\ge \widetilde V(t_{1,j+1},t_{2,k+1})-\frac{\epsilon}{2}\\ &\ge \widetilde V(t_1,t_2)-\frac{\epsilon}{2}-\frac{\epsilon}{2}\\ &=\widetilde V(t_1,t_2)-\epsilon,\end{aligned}$$

where the last inequality follows from the observation

$$\begin{aligned}&\widetilde V(t_1,t_2)-\widetilde V(t_{1,j+1},t_{2,k+1})\\ &\le \widetilde V(t_{1,j},0)-\widetilde V(t_{1,j+1},0)+\widetilde V(0,t_{2,k})-\widetilde V(0,t_{2,k+1})\\ &\le\frac{\epsilon}{2}.\end{aligned}$$

The uniform convergence result thus follows. Next we show that $P(\hat t_1\le \hat t_{0,1},\hat t_2\le \hat t_{0,2})\to 1$. To this end, we define

$$k_1=\sum_{j\in\mathcal M_1}\widehat F_{j,B}(\hat t_{0,1},0),\quad k_2=\sum_{j\in\mathcal M_1}\widehat F_{j,B}(0,\hat t_{0,2}).$$

By Assumption 5, (S18) and (S19),

$$\begin{aligned}&P(\widehat{\mathrm{FDP}}(\hat t_{01},0)<q,\widehat{\mathrm{FDP}}(0,\hat t_{02})<q)\\ &=P\left(\frac{\sum_{j\in\mathcal M_0}\widehat F_{j,B}(\hat t_{0,1},0)+k_1}{1\vee mS_{n,m}(\hat t_{0,1},0)}<q,\right.\\ &\qquad\left.\frac{\sum_{j\in\mathcal M_0}\widehat F_{j,B}(0,\hat t_{0,2})+k_2}{1\vee mS_{n,m}(0,\hat t_{0,2})}<q\right)\xrightarrow{p}1.\end{aligned}$$

Note that $\widehat{\mathrm{FDP}}(t_1,t_2)\ge\sum_{j\in\mathcal M_0}\widehat F_{j,B}(t_1,t_2)/\{1\vee mS_{n,m}(t_1,t_2)\}$. Therefore,

$$\begin{aligned}&\mathrm{FDP}(\hat t_1,\hat t_2)-\widehat{\mathrm{FDP}}(\hat t_1,\hat t_2)\\ \le&\frac{m_0Q_{n,m}(\hat t_1,\hat t_2)}{1\vee mS_{n,m}(\hat t_1,\hat t_2)}-\frac{\sum_{j\in\mathcal M_0}\widehat F_{j,B}(\hat t_1,\hat t_2)}{1\vee mS_{n,m}(\hat t_1,\hat t_2)}\\ =&\frac{m_0Q_{n,m}(\hat t_1,\hat t_2)}{1\vee mS_{n,m}(\hat t_1,\hat t_2)}-\frac{m_0\widehat Q_{n,m}(\hat t_1,\hat t_2)}{1\vee mS_{n,m}(\hat t_1,\hat t_2)}\\ &+\frac{m_0\widehat Q_{n,m}(\hat t_1,\hat t_2)}{1\vee mS_{n,m}(\hat t_1,\hat t_2)}-\frac{\sum_{j\in\mathcal M_0}\widehat F_{j,B}(\hat t_1,\hat t_2)}{1\vee mS_{n,m}(\hat t_1,\hat t_2)}\\ \le&\pi_0\epsilon/c_2+o_p(1),\end{aligned}$$

which implies that $\mathrm{FDP}(\hat t_1,\hat t_2)\le q+\pi_0\epsilon/c_2+o_p(1)$. As ϵ can be arbitrarily small, the conclusion follows. □

Remark 4. We note that

$$\begin{aligned}&|\widehat Q_{n,m}(t_1,t_2)-Q_{n,m}(t_1,t_2)|\\ \le&\sup_{j\in\mathcal M_0}\left|\int \mathbf 1\{T_j^M(\widetilde{\mathbf x},\widetilde{\mathbf Y}_j)\ge t_1, T_j^C(\widetilde{\mathbf x},\widetilde{\mathbf Y}_j,\widetilde{\mathbf Z})\ge t_2\}\left(d\widehat{\mathbb P}(\widetilde{\mathbf x}|\widetilde{\mathbf Z})-d\mathbb P(\widetilde{\mathbf x}|\widetilde{\mathbf Z})\right)\right|\\ \le& d_{\mathrm{TV}}\left(\prod_{i=1}^n\widehat{\mathbb P}_{\mathbf X|\mathbf Z}(\mathbf x_i|\mathbf Z_i),\prod_{i=1}^n\mathbb P_{\mathbf X|\mathbf Z}(\mathbf x_i|\mathbf Z_i)\right)\\ \le&1-\prod_{i=1}^n\left\{1-d_{\mathrm H}^2(\widehat{\mathbb P}_{\mathbf X|\mathbf Z}(\mathbf x_i|\mathbf Z_i),\mathbb P_{\mathbf X|\mathbf Z}(\mathbf x_i|\mathbf Z_i))\right\},\end{aligned}$$

where d_{TV} and $d_{\mathrm H}$ denote the total variation and the Hellinger distances respectively. A sufficient condition for the above bound to go to zero is that $\max_i d_{\mathrm H}^2(\widehat{\mathbb P}_{\mathbf X|\mathbf Z}(\mathbf x_i|\mathbf Z_i),\mathbb P_{\mathbf X|\mathbf Z}(\mathbf x_i|\mathbf Z_i))=o_p(n^{-1/2})$, which has been considered in [2].

Proof of Corollary SC1 Recall that $\mathrm{TP}_{2\mathrm d}\ge\frac{1-q_2}{1-q_1}\mathrm{TP}_{1\mathrm d}$. By (S17) in the proof of Theorem 1, $\mathbb P(q_2\le q+\epsilon)\to 1$. As $q_1\ge 0$, the conclusion follows. □

S7. Details on Asymptotic Power Analysis

We perform an asymptotic power analysis by comparing the asymptotic power of 2dFDR+ with that of the associated 1d procedure. For $(t_1,t_2)\in\mathbb R^+\times\mathbb R^+$, define $\widetilde K(t_1,t_2)$ as

$$\widetilde K(t_1,t_2)=\frac{\widetilde S(t_1,t_2)-\pi_0\widetilde V(t_1,t_2)}{(1-\pi_0)},\qquad\text{(S20)}$$

which can be considered as the limiting power process. Assume that

$$\sup_{t_1\le t_{0,1},t_2\le t_{0,2}}\left|\frac{1}{m_1}\sum_{j\in\mathcal M_1}\bar F_{j,B}(t_1,t_2)-\widetilde U(t_1,t_2)\right|\to^p 0$$

for some non-negative function $\widetilde U$. Let

$$\widetilde{\mathrm{FDP}^\infty}(t_1,t_2)=\frac{\pi_0\widetilde V(t_1,t_2)+(1-\pi_0)\widetilde U(t_1,t_2)}{\widetilde S(t_1,t_2)},$$

which is the limiting process for $\widetilde{\mathrm{FDP}}(t_1,t_2)$ in view of the derivations in Section S6. To understand the power behavior of 2dFDR+ and the associated 1d procedure, we consider the following (infeasible) procedures based on the above limiting processes:

$$\begin{aligned}\text{Limiting 2dFDR+:}\quad &(t^*_{1,2\mathrm d},t^*_{2,2\mathrm d})=\mathop{\arg\max}_{(t_1,t_2)\in\mathbb R^+\times\mathbb R^+}\widetilde S(t_1,t_2)\\ &\text{subject to}\quad \widetilde{\mathrm{FDP}^\infty}(t_1,t_2)\le q,\\ \text{Limiting 1dFDR:}\quad &t^*_{1\mathrm d}=\mathop{\arg\max}_{t\in\mathbb R^+}\widetilde S(0,t)\\ &\text{subject to}\quad \widetilde{\mathrm{FDP}^\infty}(0,t)\le q.\end{aligned}$$

As $(0,t^*_{1\mathrm d})$ is a feasible point of the optimization problem in limiting 2dFDR+, we must have

$$\widetilde S(t^*_{1,2\mathrm d},t^*_{2,2\mathrm d})\ge\widetilde S(0,t^*_{1\mathrm d}).\qquad\text{(S21)}$$

Assume that $\widetilde{\mathrm{FDP}^\infty}(t_1,t_2)$ is a continuous function of (t_1,t_2). Then we have

$$\widetilde{\mathrm{FDP}^\infty}(t^*_{1,2\mathrm d},t^*_{2,2\mathrm d})=\widetilde{\mathrm{FDP}^\infty}(0,t^*_{1\mathrm d})=q,$$

as otherwise one can lower the values of (t_1, t_2) to increase the value of the objective function $\widetilde{S}$. Some algebra yields that

$$\frac{(1-\pi_0)\{\widetilde{K}(t^*_{1,2d}, t^*_{2,2d}) - \widetilde{U}(t^*_{1,2d}, t^*_{2,2d})\}}{\widetilde{S}(t^*_{1,2d}, t^*_{2,2d})} = \frac{(1-\pi_0)\{\widetilde{K}(0, t^*_{1d}) - \widetilde{U}(0, t^*_{1d})\}}{\widetilde{S}(0, t^*_{1d})} = 1 - q.$$

By (S21), we have

$$\widetilde{K}(t^*_{1,2d}, t^*_{2,2d}) \geq \frac{1 - q + (1-\pi_0)\widetilde{U}(t^*_{1,2d}, t^*_{2,2d})/\widetilde{S}(t^*_{1,2d}, t^*_{2,2d})}{1 - q + (1-\pi_0)\widetilde{U}(0, t^*_{1d})/\widetilde{S}(0, t^*_{1d})}\widetilde{K}(0, t^*_{1d}) \geq (1-q)\widetilde{K}(0, t^*_{1d}).$$

Comparing to the result in Corollary SC1, we derive two terms $(1 - \pi_0)\widetilde{U}(t^*_{1,2d}, t^*_{2,2d})/\widetilde{S}(t^*_{1,2d}, t^*_{2,2d})$ and $(1 - \pi_0)\widetilde{U}(0, t^*_{1d})/\widetilde{S}(0, t^*_{1d})$ that determine the power improvement. In the worst-case scenario, $\widetilde{K}(t^*_{1,2d}, t^*_{2,2d}) \geq (1-q)\widetilde{K}(0, t^*_{1d})$, which again suggests that the power loss is at most q.

S8. DGPs in the simulation studies

We provide the specific data generating processes (DGPs) considered in Sections 4.3 and S9:

1. $Y_j = \alpha_j X + \beta_j Z + \epsilon_j$ and $X \sim N(\rho Z, 1)$, where $Z \sim N(0, 1)$;
2. $Y_j = \alpha_j X^3 + \beta_j e^Z + \epsilon_j$ and $X \sim N(\rho Z^2, 1)$, where $Z \sim N(0, 1)$;
3. $Y_j = \alpha_j X^3 + \beta_j Z^3 + \epsilon_j$ and $X \sim N(\rho(Z + Z^2), 1)$, where $Z \sim N(0, 1)$;
4. $Y_j = \alpha_j(X + |X^3|) + \beta_j e^Z + \epsilon$ and $X \sim N(\rho(Z + Z^2), 1)$, where $Z \sim N(0, 1)$;
5. $Y_j = \alpha_j e^X + \beta_j Z + \epsilon_j$ and $X \sim \text{Bernoulli}((1 + e^{-\rho Z})^{-1})$, where $Z \sim N(0, 1)$;
6. $Y_j = \alpha_j e^X + \beta_j e^Z + \epsilon_j$ and $X \sim \text{Bernoulli}((1 + e^{-\rho Z})^{-1})$, where $Z \sim N(0, 1)$;
7. $Y_j = \alpha_j e^X + \beta_j Z^2 + \epsilon_j$ and $X \sim \text{Bernoulli}((1 + e^{-\rho Z})^{-1})$, where $Z \sim N(0, 1)$;
8. $Y_j = \alpha_j X + \beta_j Z + \epsilon_j$ and $X \sim \text{Bernoulli}((1 + e^{-\rho Z})^{-1})$, where $Z \sim \text{Bernoulli}(0.7)$;
9. $Y_j \sim \text{Bernoulli}((1 + e^{-f_j(X,Z)})^{-1})$, where $f_j(X, Z) = \alpha_j X + \beta_j Z$, $X \sim N(\rho Z, 1)$ and $Z \sim N(0, 1)$;
10. $Y_j \sim \text{Poisson}(\lambda_j)$, where $\log \lambda_j = \alpha_j X + \beta_j Z$ with $X \sim N(\rho Z, 1)$ and $Z \sim N(0, 1)$;
11. $Y_j \sim \text{Negative Binomial}(\text{size} = 3, \mu_j = e^{f_j(X,Z)})$, where $f_j(X, Z) = \alpha_j X + \beta_j Z$, $X \sim N(\rho Z, 1)$ and $Z \sim N(0, 1)$.

S9. Additional simulation results

1. *Linear/nonlinear models with discrete X and continuous Z.* In particular, we generate
$$X \sim \text{Bernoulli}\left(\frac{e^{\rho Z}}{1 + e^{\rho Z}}\right),$$
where $Z \sim N(0, 1)$. Models 5-7 explore this setup. In this case, we generate $X_{i,b}$ through a fitted logistic regression model using Z as the predictor. We report the FDR and power for MS-1dFDR, RV-1dFDR, 2dFDR, MS-2dFDR+ and RV-2dFDR+ as described in Section 5.2 in Figures S4-S6. HSIC-2dFDR+ and HSIC-1dFDR are not used in this data generating setup because for binary variables, HSIC is not efficient and the bandwidth parameter is not well-defined. the empirical FDR is well controlled for 2dFDR+ even when the confounding effect is strong. 2dFDR suffers from moderate FDR inflation (e.g Figure S5a) in some instances, e.g., in the case of strong confounding. Not surprisingly, the 2d procedure is significantly more powerful than the corresponding 1d version. Moreover, the RV-based methods generally make more true rejections compared to the HSIC-based methods.
2. *Linear models with discrete X and Z.* We consider the linear model
$$Y_j = \alpha_j X + \beta_j Z + \epsilon_j,$$
where
$$X \sim \text{Bernoulli}\left(\frac{e^{\rho Z}}{1 + e^{\rho Z}}\right) \quad \text{and} \quad Z \sim \text{Bernoulli}(0.7).$$
The results for MS-1dFDR, RV-1dFDR, MS-2dFDR+ and RV-2dFDR+ are reported in Figure S7. As seen from Figure S7, all the approaches have empirical FDR under control. When the degree of confounding is high, 2dFDR+ delivers higher power than 1dFDR does.
3. *Count response.* We consider the Poisson model
$$Y_j \sim \text{Poisson}(\lambda_j), \quad \log \lambda_j = \alpha_j X + \beta_j Z,$$
with $X \sim N(\rho Z, 1)$ and $Z \sim N(0, 1)$. We implement the MS-1dFDR, RV-1dFDR, MS-2dFDR+ and RV-2dFDR+, and report the results in Figure S8. Additionally, we consider the negative binomial regression model
$$Y_j \sim \text{Negative Binomial}(\text{size} = 3, \mu_j = e^{f_j(X,Z)})$$
where $f_j(X, Z) = \alpha_j X + \beta_j Z$ for $X \sim N(\rho Z, 1)$ and $Z \sim N(0, 1)$. We implement the MS-1dFDR, RV-1dFDR, MS-2dFDR+ and RV-2dFDR+, and report the results in Figure S9. For binary and count responses the original 2dFDR is not applicable, and hence only 1dFDR and 2dFDR+ have been compared in the simulations. As seen from Figures S8 and S9, all the methods provide reliable FDR control. 2dFDR+ produces significant power improvement over the 1dFDR methods.
4. FWER control: We investigate the finite sample performance of 2dFWER+ and its corresponding 1d version. In Figures S15-S16, we report the empirical FWER and power of 2dFWER+ and 1dFWER for both the linear and nonlinear models. In either case, the empirical FWER is well controlled for both methods. The 2d procedure again produces higher power than the 1d version, especially for stronger confounders.
5. Global null: We examine the performance of 2dFDR, RV-2dFDR+, HSIC-2dFDR+, RV-1dFDR and HSIC-1dFDR under the global null. Specifically, we consider the model $Y_j = \beta_j Z$, where $X \sim N(\rho Z, 1)$ and $Z \sim N(0, 1)$. None of the methods produced any rejections for all degrees of confounding.
6. Dependent errors: To evaluate the impact of dependence on the methods' performance, we consider the model: $Y_j = \alpha_j e^X + \beta_j e^Z + \epsilon_j$ where $\epsilon_j = 0.7\epsilon_{j-1} + e_j$ and $X \sim N(\rho(Z + Z^2), 1)$ with $Z \sim N(0, 1)$ and $\{e_j\}_{j=1}^m$ being a white noise process. The results are summarized in Figure S10. Overall, 2dFDR+ is robust to the AR(1)

type dependence with reliable FDR control and reasonable power.

7. Separating the effects of densities of the signal of interest and the confounder signal: In all preceding simulations, the density of the signal of interest and the confounding signal had been kept at the same level—weak, moderate or strong. In this simulation setup, we attempt to tease apart the effects of the two types of signals.

   a. First, we fix the density of the signal of interest at the 10% level and vary the density of the confounding signal through weak, moderate, and strong. The associated plots are given in Figure S11 and Figure S13, corresponding to linear and non-linear DGPs respectively.
   b. Next, we fix the density of the confounding signal to 10% and vary the density of the signal of interest through weak, moderate, and strong. The associated plots are in Figure S12 and Figure S14, corresponding to linear and non-linear DGPs respectively.

   In both the linear and the non-linear DGPs, we find that varying the density of the signal of interest while keeping the density of the confounding variable constant is displaying a starker difference (increase) in the power as the densities are increased.

To sum up, the proposed 2dFDR+ provides reliable FDR control for all the simulation settings even when the degree of confounding is strong because 2dFDR+ explicitly models the relationship between X and Z. The 2d procedure delivers more rejections compared to the 1d counterpart, and the larger number of rejections typically translates into a higher detection power for the 2d methods. We also see that RV-2dFDR+ provides the best power in many simulation settings. As the (conditional) RV coefficients are calculated based on spline-transformed covariates and confounding factors, RV-2dFDR+ can capture the nonlinearity between Y and (X, Z) and X and Z in many cases.

S10. Microbiome data: Binary Outcomes

We consider the microbiome data analyzed in Section 6 of the main paper. The abundance data of the 174 OTUs were converted into presence/absence data after rarefaction to the minimal sequencing depth (since presence/absence depends on the sequencing depth strongly, rarefaction removes the confounding effect due to sequence depth). Because X, Y, and Z are all categorical (specifically, binary) in this case, for the conditional statistic, i.e., T^C, the Mantel Haenszel statistic was used. For the marginal statistic, i.e., T^M, the Pearson's chi-square statistic was used. Note that the original 2dFDR in [13] is not applicable in this case as the outcomes are binary. As shown in Figure S1, for all levels of FDR, the BH procedure makes no rejections, and overall, the 2dFDR+ procedure makes a higher number of rejections compared to the corresponding 1dFDR procedure.

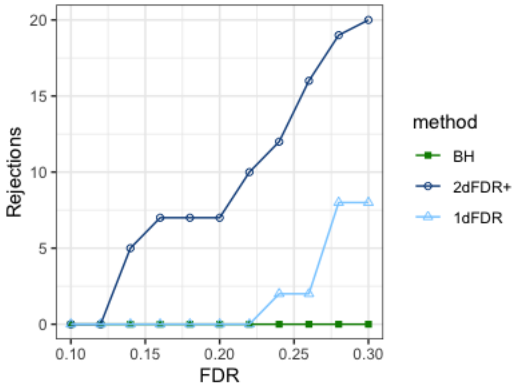

Fig. S1. Number of Rejections versus FDR for different methods for smoking microbiome data, where the continuous abundance data were transformed into presence/absence (binary) data.

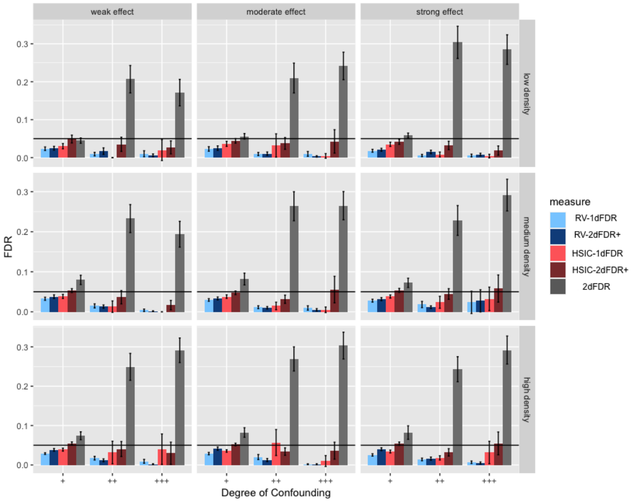

(a) FDR

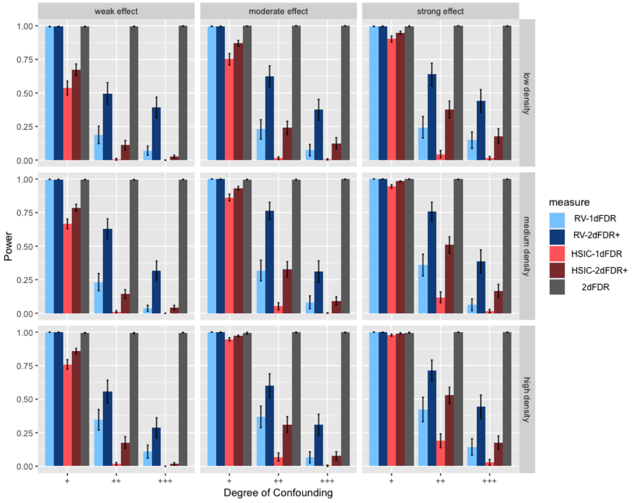

(b) Power

Fig. S2. Empirical FDR and power for HSIC-1dFDR, RV-1dFDR, 2dFDR, HSIC-2dFDR+, RV-2dFDR+ under the model $Y_j = \alpha_j X^3 + \beta_j Z^3 + \epsilon_j$ and $X \sim N(\rho(Z + Z^2), 1)$, where $Z \sim N(0, 1)$. Error bars represent the 95% CIs and the horizontal line in (a) indicates the target FDR level of 0.05.

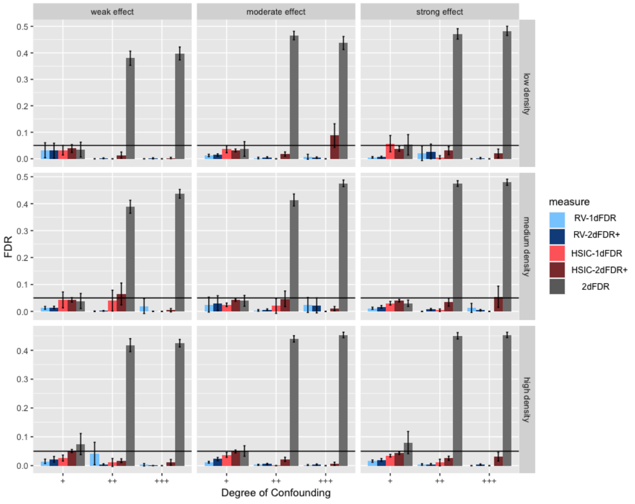

(a) FDR

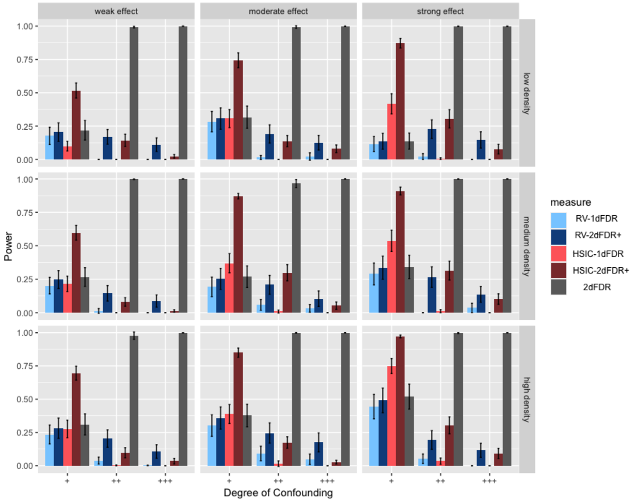

(b) Power

Fig. S3. Empirical FDR and power for HSIC-1dFDR, RV-1dFDR, 2dFDR, HSIC-2dFDR+, RV-2dFDR+ under the model $Y_j = \alpha_j(X + |X^3|) + \beta_j e^Z + \epsilon$ and $X \sim N(\rho(Z + Z^2), 1)$, where $Z \sim N(0, 1)$. Error bars represent the 95% CIs and the horizontal line in (a) indicates the target FDR level of 0.05.

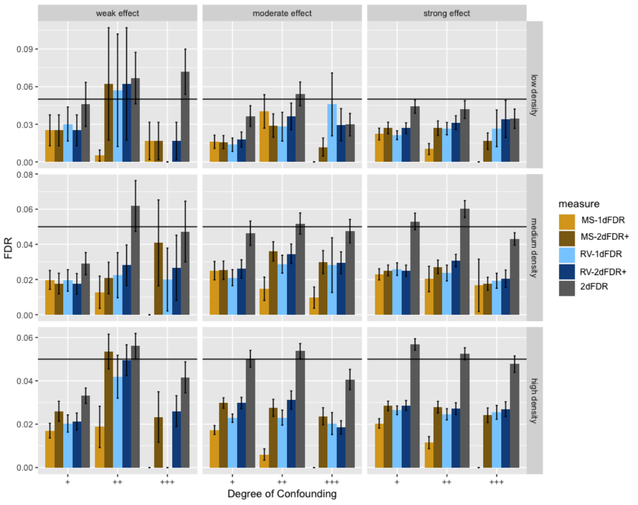

(a) FDR

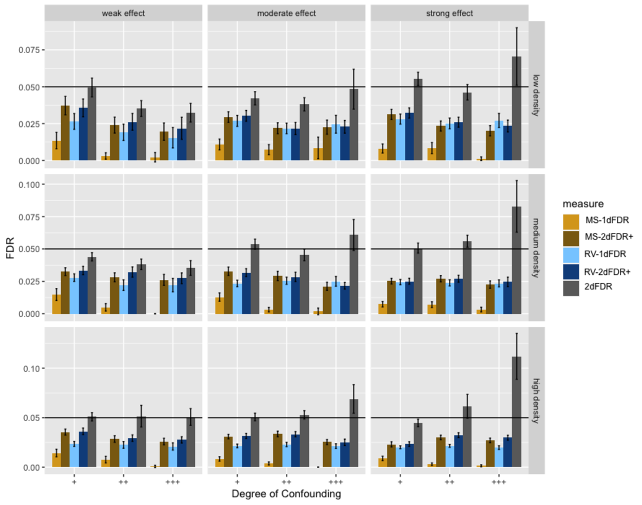

(a) FDR

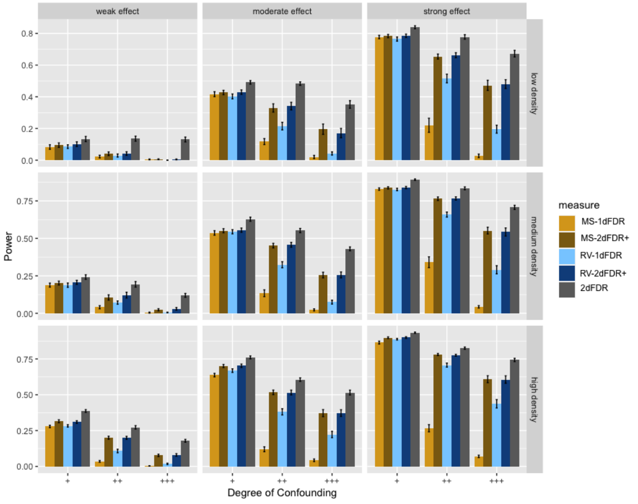

(b) Power

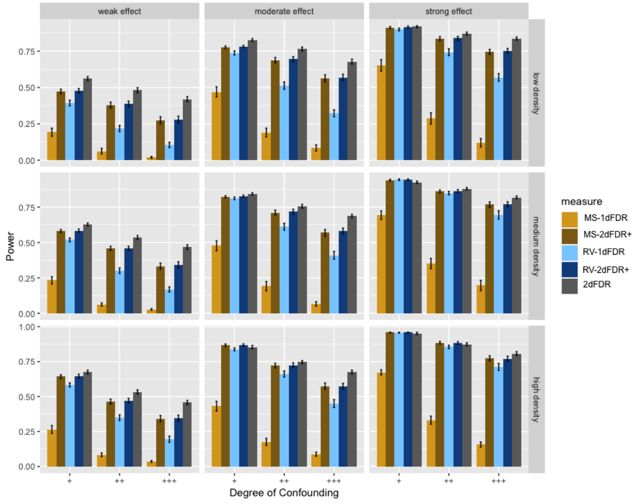

(b) Power

Fig. S4. Empirical FDR and power for MS-1dFDR, RV-1dFDR, 2dFDR, MS-2dFDR+, RV-2dFDR+ under the model $Y_j = \alpha_j e^X + \beta_j Z + \epsilon_j$ and $X \sim \text{Bernoulli}((1 + e^{-\rho Z})^{-1})$, where $Z \sim N(0, 1)$. Error bars represent the 95% CIs and the horizontal line in (a) indicates the target FDR level of 0.05.

Fig. S5. Empirical FDR and power for MS-1dFDR, RV-1dFDR, 2dFDR, MS-2dFDR+, RV-2dFDR+ under the model $Y_j = \alpha_j e^X + \beta_j e^Z + \epsilon_j$ and $X \sim \text{Bernoulli}((1 + e^{-\rho Z})^{-1})$, where $Z \sim N(0, 1)$. Error bars represent the 95% CIs and the horizontal line in (a) indicates the target FDR level of 0.05.

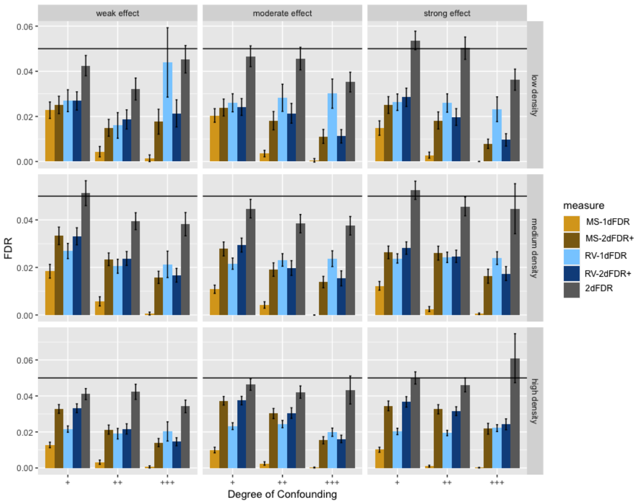

(a) FDR

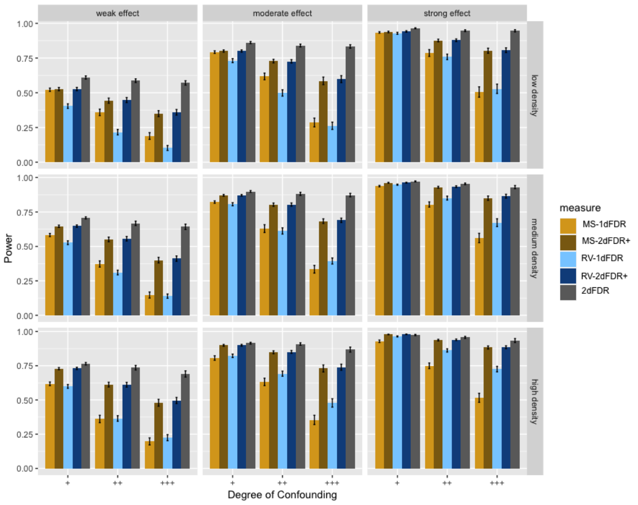

(b) Power

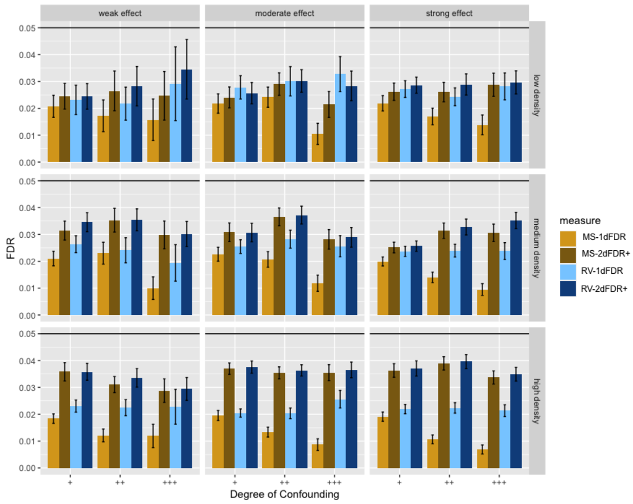

(a) FDR

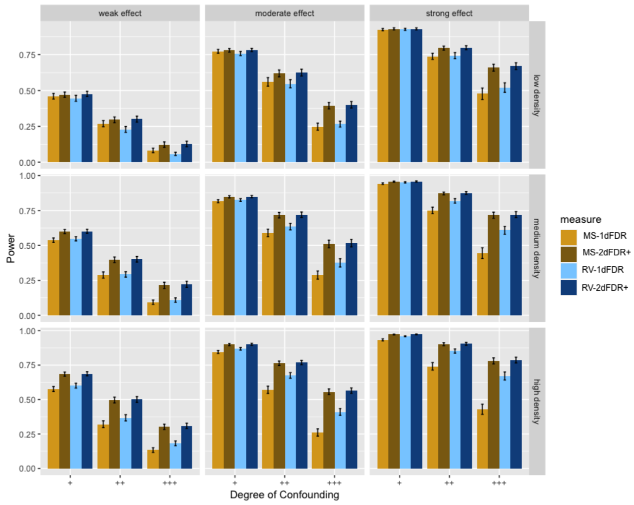

(b) Power

Fig. S6. Empirical FDR and power for MS-1dFDR, RV-1dFDR, 2dFDR, MS-2dFDR+, RV-2dFDR+ under the model $Y_j = \alpha_j e^X + \beta_j Z^2 + \epsilon_j$ and $X \sim \text{Bernoulli}((1+e^{-\rho Z})^{-1})$, where $Z \sim N(0,1)$. Error bars represent the 95% CIs and the horizontal line in (a) indicates the target FDR level of 0.05.

Fig. S7. Empirical FDR and power for MS-1dFDR, RV-1dFDR, MS-2dFDR+, RV-2dFDR+ under the model $Y_j = \alpha_j X + \beta_j Z + \epsilon_j$ and $X \sim \text{Bernoulli}((1+e^{-\rho Z})^{-1})$, where $Z \sim \text{Bernoulli}(0.7)$. Error bars represent the 95% CIs and the horizontal line in (a) indicates the target FDR level of 0.05.

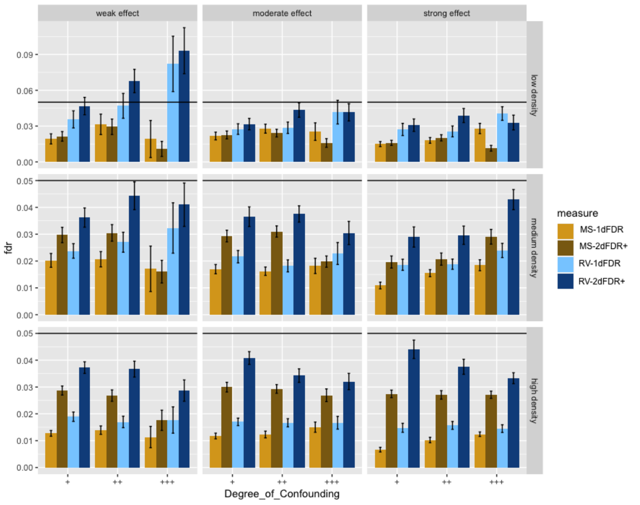

(a) FDR

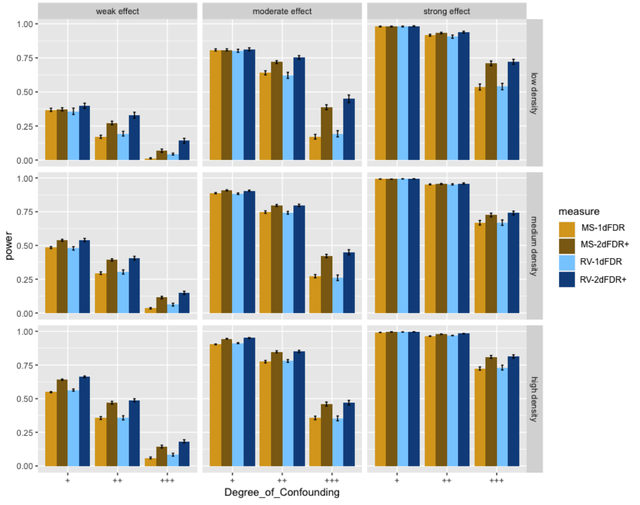

(b) Power

Fig. S8. Empirical FDR and power for MS-1dFDR, RV-1dFDR, MS-2dFDR+, RV-2dFDR+ under the model $Y_j \sim \text{Poisson}(\lambda_j)$, where $\log \lambda_j = \alpha_j X + \beta_j Z$ with $X \sim N(\rho Z, 1)$ and $Z \sim N(0, 1)$. Error bars represent the 95% CIs and the horizontal line in (a) indicates the target FDR level of 0.05.

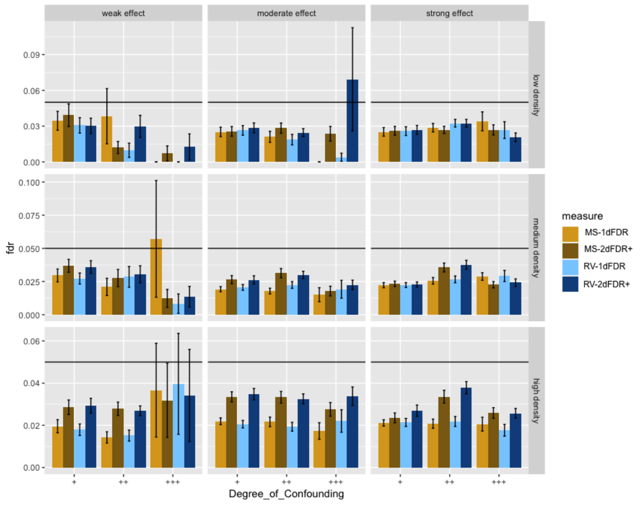

(a) FDR

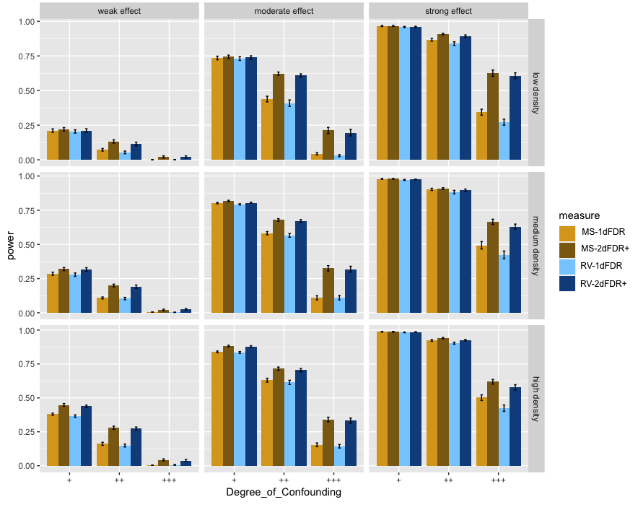

(b) Power

Fig. S9. Empirical FDR and power for MS-1dFDR, RV-1dFDR, MS-2dFDR+, RV-2dFDR+ under the model $Y_j \sim \text{Negative Binomial}(\text{size} = 3, \mu_j = e^{f_j(X,Z)})$, where $f_j(X, Z) = \alpha_j X + \beta_j Z$, $X \sim N(\rho Z, 1)$ and $Z \sim N(0, 1)$. Error bars represent the 95% CIs and the horizontal line in (a) indicates the target FDR level of 0.05.

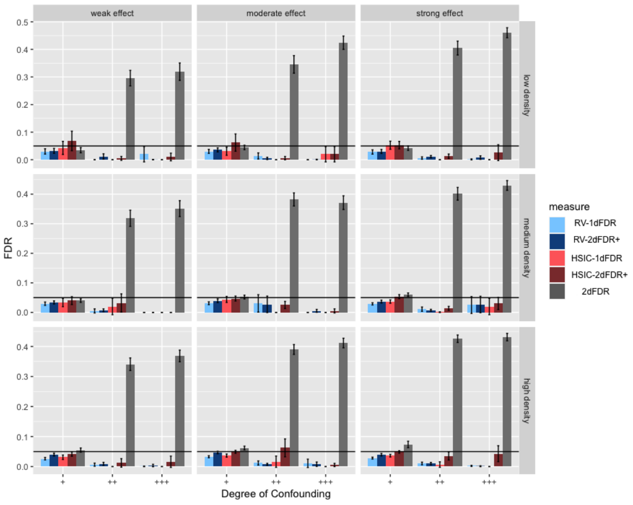

(a) FDR

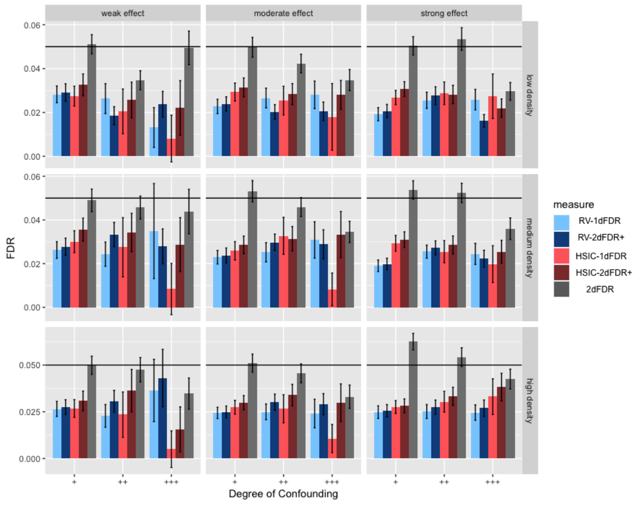

(a) FDR

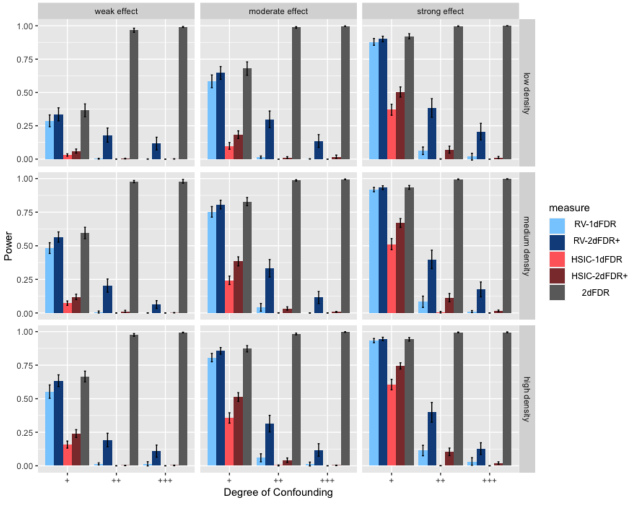

(b) Power

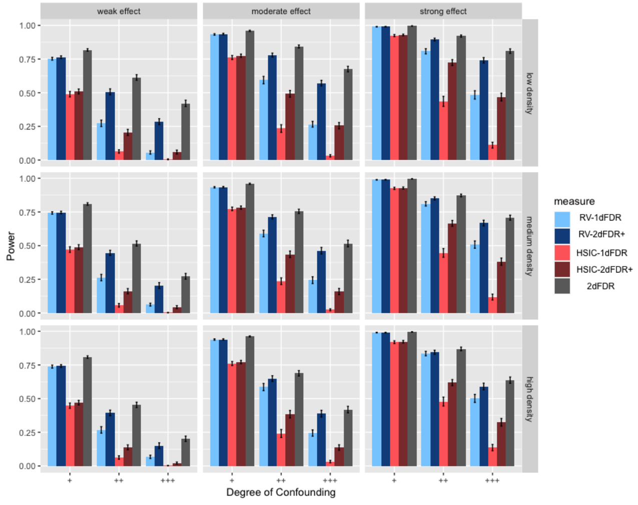

(b) Power

Fig. S10. Empirical FDR and power for HSIC-1dFDR, RV-1dFDR, 2dFDR, HSIC-2dFDR+, RV-2dFDR+ under the model $Y_j = \alpha_j e^X + \beta_j e^Z + \epsilon_j$, where ϵ_j follows an AR(1) model with the AR(1) coefficient being 0.7, $X \sim N(\rho(Z + Z^2), 1)$ and $Z \sim N(0, 1)$ Error bars represent the 95% CIs and the horizontal line in (a) indicates the target FDR level of 0.05.

Fig. S11. Empirical FDR and power for HSIC-1dFDR, RV-1dFDR, 2dFDR, HSIC-2dFDR+, RV-2dFDR+ under the model $Y_j = \alpha_j X + \beta_j Z + \epsilon_j$ where $X \sim N(\rho Z, 1)$ and $Z \sim N(0, 1)$. The signal density of α_j has been fixed at 10 % while the signal density of β_j has been varied through 1%, 5% and 10%. Error bars represent the 95% CIs and the horizontal line in (a) indicates the target FDR level of 0.05.

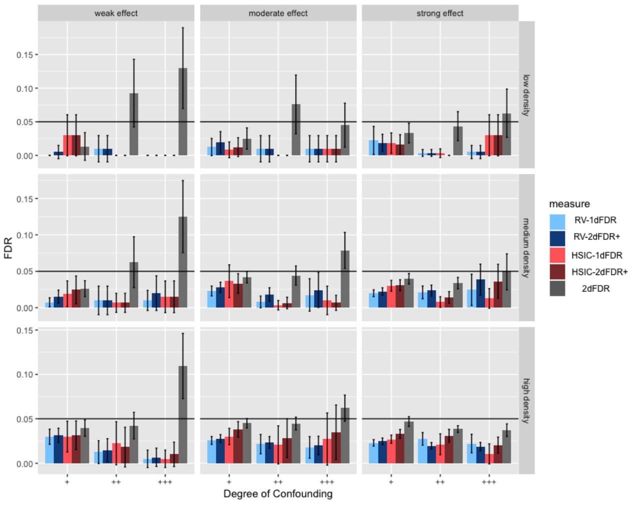

(a) FDR

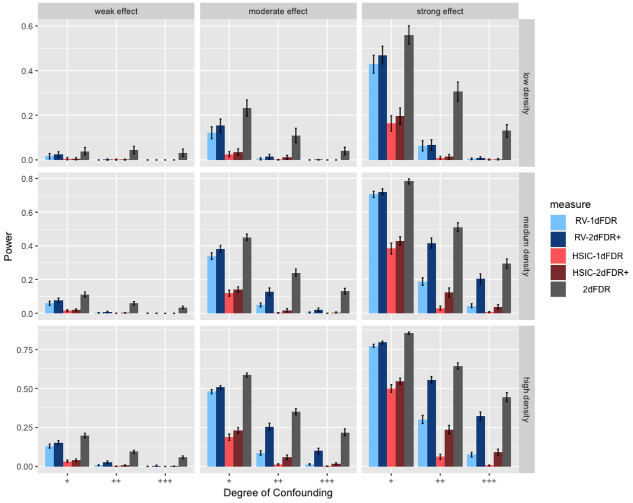

(b) Power

Fig. S12. Empirical FDR and power for HSIC-1dFDR, RV-1dFDR, 2dFDR, HSIC-2dFDR+, RV-2dFDR+ under the model $Y_j = \alpha_j X + \beta_j Z + \epsilon_j$ where $X \sim N(\rho Z, 1)$ and $Z \sim N(0, 1)$. The signal density of β_j has been fixed at 10 % while the signal density of α_j has been varied through 1%, 5% and 10%. Error bars represent the 95% CIs and the horizontal line in (a) indicates the target FDR level of 0.05.

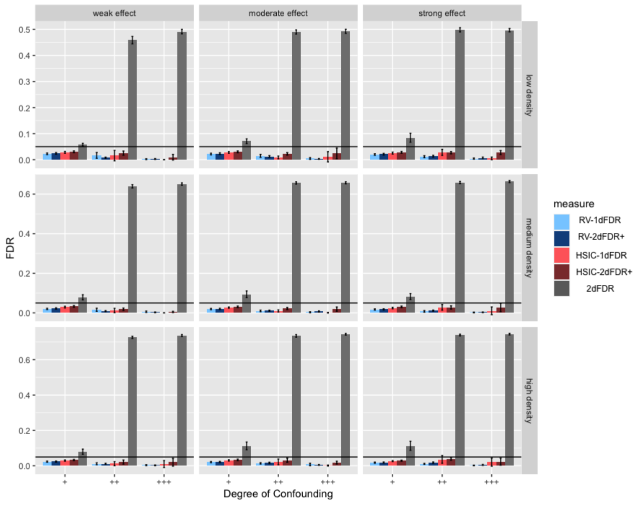

(a) FDR

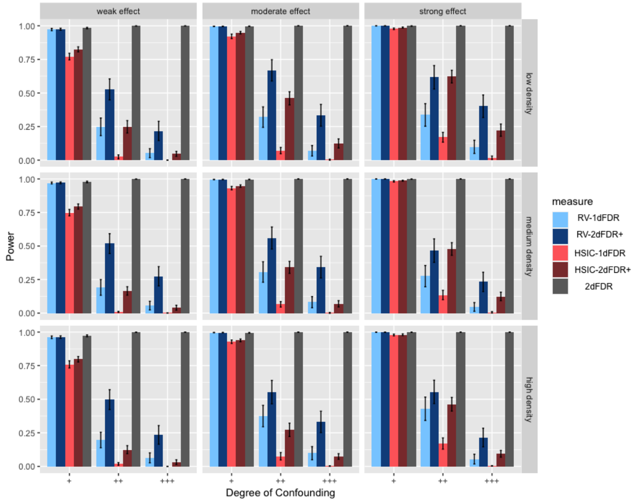

(b) Power

Fig. S13. Empirical FDR and power for HSIC-1dFDR, RV-1dFDR, 2dFDR, HSIC-2dFDR+, RV-2dFDR+ under the model $Y_j = \alpha_j e^X + \beta_j Z^2 + \epsilon_j$ where $X \sim N(\rho Z^2, 1)$ and $Z \sim N(0, 1)$. The signal density of α_j has been fixed at 10 % while the signal density of β_j has been varied through 1%, 5% and 10%. Error bars represent the 95% CIs and the horizontal line in (a) indicates the target FDR level of 0.05.

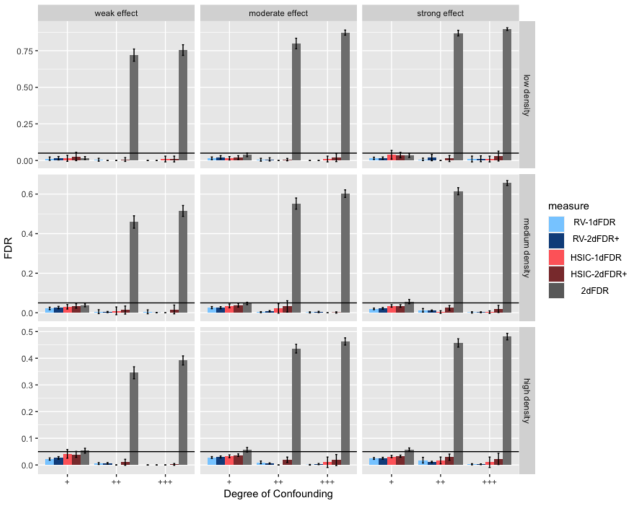

(a) FDR

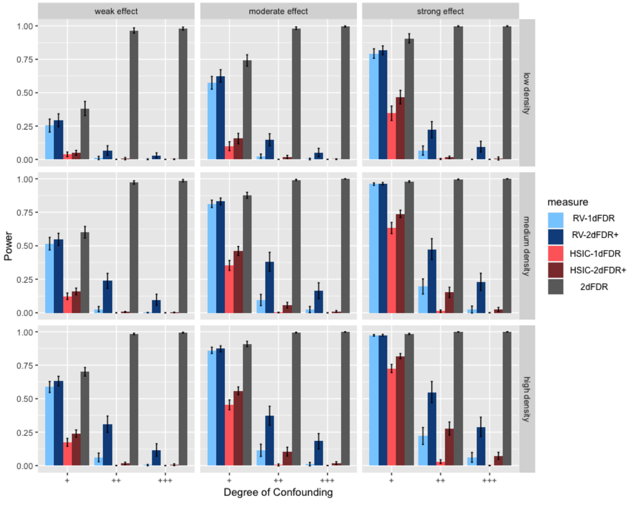

(b) Power

Fig. S14. Empirical FDR and power for HSIC-1dFDR, RV-1dFDR, 2dFDR, HSIC-2dFDR+, RV-2dFDR+ under the model $Y_j = \alpha_j e^X + \beta_j Z^2 + \epsilon_j$ where $X \sim N(\rho Z^2, 1)$ and $Z \sim N(0, 1)$. The signal density of β_j has been fixed at 10 % while the signal density of α_j has been varied through 1%, 5% and 10%. Error bars represent the 95% CIs and the horizontal line in (a) indicates the target FDR level of 0.05.

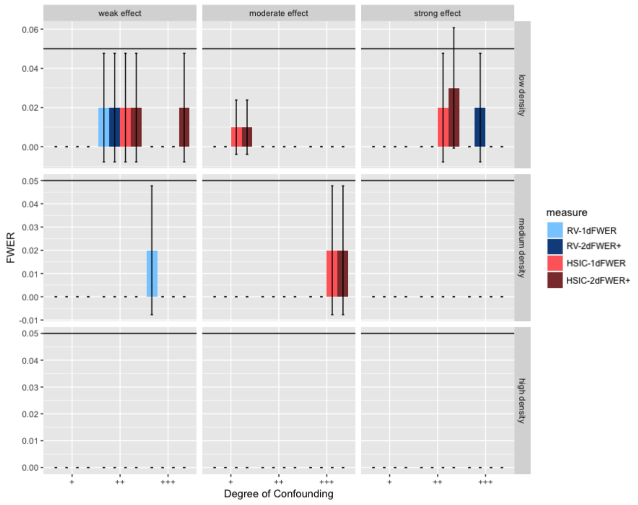

(a) FWER

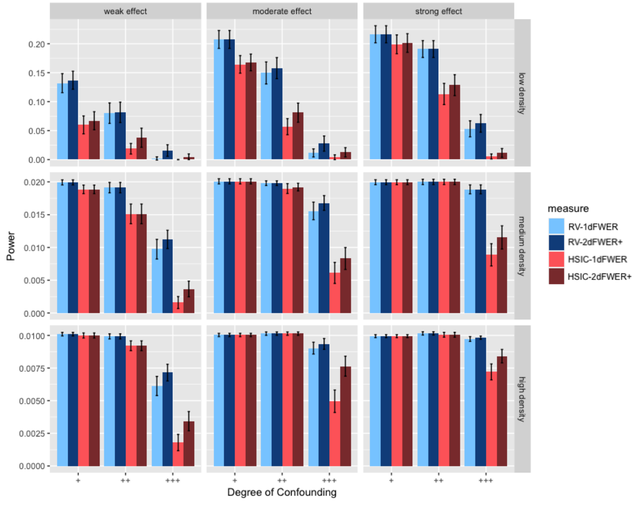

(b) Power

Fig. S15. Empirical FWER and power for HSIC-1dFWER, RV-1dFWER, HSIC-2dFWER+, RV-2dFWER+ under the model $Y_j = \alpha_j X + \beta_j Z + \epsilon_j$, where $X \sim N(\rho Z, 1)$ and $Z \sim N(0, 1)$. Error bars represent the 95% CIs and the horizontal line in (a) indicates the target FWER level of 0.05.

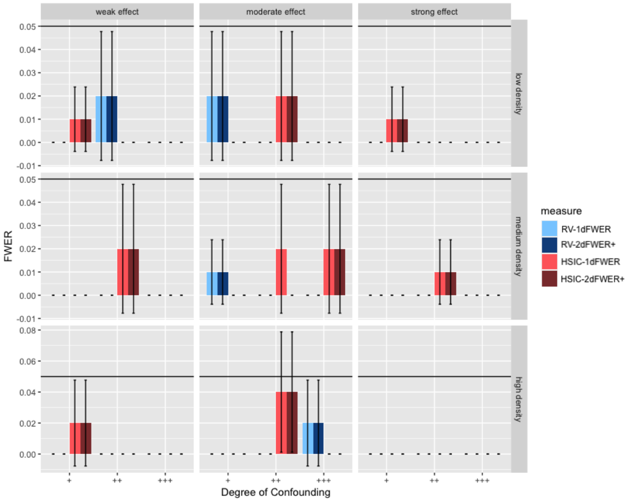

(a) FWER

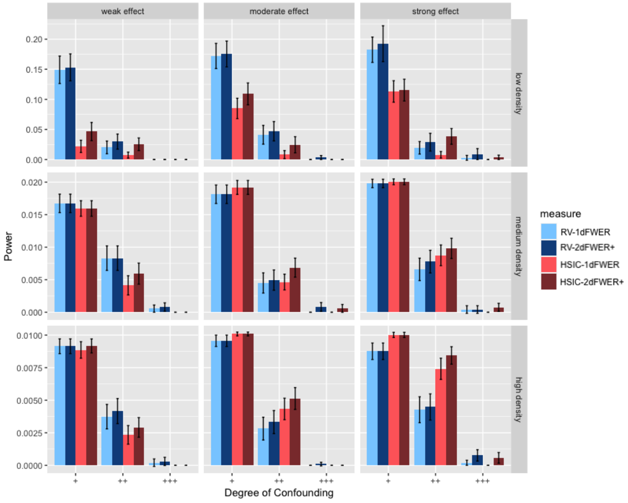

(b) Power

Fig. S16. Empirical FWER and power for HSIC-1dFWER, RV-1dFWER, HSIC-2dFWER+, RV-2dFWER+ under the model $Y_j = \alpha_j e^X + \beta_j Z^2 + \epsilon_j$, where $X \sim N(\rho Z^2, 1)$ and $Z \sim N(0, 1)$. Error bars represent the 95% CIs and the horizontal line in (a) indicates the target FWER level of 0.05.

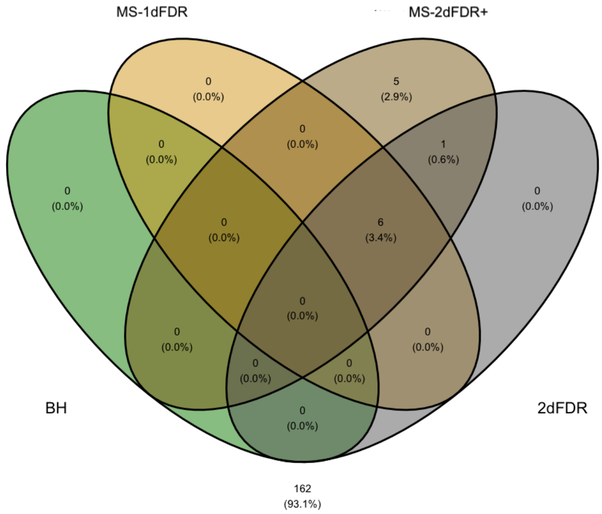

Fig. S17. Venn diagram of features identified by different methods for smoking microbiome data

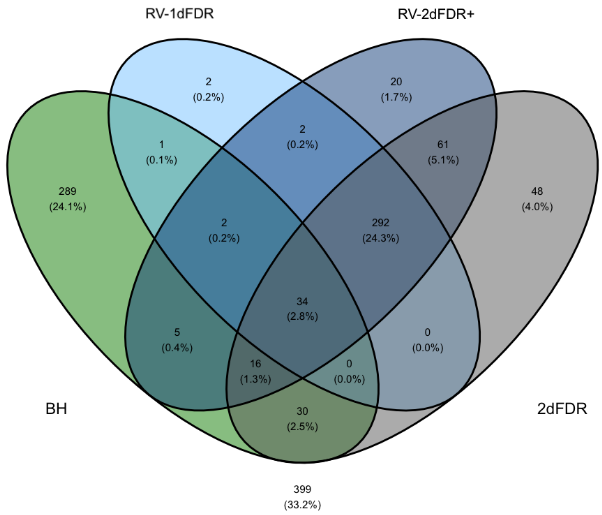

Fig. S18. Venn diagram of features identified by different methods for metabolomics data

References

Wicher Bergsma and Angelos Dassios. A consistent test of independence based on a sign covariance related to kendall's tau. *Bernoulli*, 20(2):1006–1028, 2014.

TB Berrett and RJ Samworth. Nonparametric independence testing via mutual information. *Biometrika*, 106(3):547–566, 2019.

Hongyuan Cao, Jun Chen, and Xianyang Zhang. Optimal false discovery rate control for large scale multiple testing with auxiliary information. *The Annals of Statistics*, 50(2):807–857, 2022.

Anthony Christopher Davison and David Victor Hinkley. *Bootstrap methods and their application.* Number 1. Cambridge university press, 1997.

Arthur Gretton, Olivier Bousquet, Alex Smola, and Bernhard Schölkopf. Measuring statistical dependence with hilbert-schmidt norms. In *International conference on algorithmic learning theory*, pages 63–77. Springer, 2005.

Arthur Gretton, Kenji Fukumizu, Choon Hui Teo, Le Song, Bernhard Schölkopf, Alexander J Smola, et al. A kernel statistical test of independence. In *Nips*, volume 20, pages 585–592. Citeseer, 2007.

Mehdi Mirza and Simon Osindero. Conditional generative adversarial nets. *arXiv preprint arXiv:1411.1784*, 2014.

Michael Naaman. On the tight constant in the multivariate dvoretzky–kiefer–wolfowitz inequality. *Statistics & Probability Letters*, 173:109088, 2021.

Bharath K Sriperumbudur, Kenji Fukumizu, and Gert RG Lanckriet. Universality, characteristic kernels and rkhs embedding of measures. *Journal of Machine Learning Research*, 12(7), 2011.

John D Storey, Jonathan E Taylor, and David Siegmund. Strong control, conservative point estimation and simultaneous conservative consistency of false discovery rates: a unified approach. *Journal of the Royal Statistical Society: Series B (Statistical Methodology)*, 66(1):187–205, 2004.

Gábor J Székely, Maria L Rizzo, and Nail K Bakirov. Measuring and testing dependence by correlation of distances. *The annals of statistics*, 35(6):2769–2794, 2007.

Anderson M Winkler, Gerard R Ridgway, Matthew A Webster, Stephen M Smith, and Thomas E Nichols. Permutation inference for the general linear model. *Neuroimage*, 92:381–397, 2014.

Sangyoon Yi, Xianyang Zhang, Lu Yang, Jinyan Huang, Yuanhang Liu, Chen Wang, Daniel J Schaid, and Jun Chen. 2dfdr: a new approach to confounder adjustment substantially increases detection power in omics association studies. *Genome biology*, 22(1):1–18, 2021.

Kun Zhang, Jonas Peters, Dominik Janzing, and Bernhard Schölkopf. Kernel-based conditional independence test and application in causal discovery. *arXiv preprint arXiv:1202.3775*, 2012.

Xingyu Zhou, Yuling Jiao, Jin Liu, and Jian Huang. A deep generative approach to conditional sampling. *Journal of the American Statistical Association*, pages 1–12, 2022.
